# Developmental-status-aware transcriptional decomposition establishes a cell state panorama of human cancers

**DOI:** 10.1186/s13073-024-01393-6

**Published:** 2024-10-28

**Authors:** Yikai Luo, Han Liang

**Affiliations:** 1 Graduate Program in Quantitative and Computational Biosciences, Baylor College of Medicine, Houston, TX 77030 USA; 2 Department of Bioinformatics and Computational Biology, The University of Texas MD Anderson Cancer Center, Houston, TX 77030 USA; 3 Present Address: Division of Rheumatology, Department of Medicine, University of California, San Francisco, San Francisco, CA 94143 USA; 4 Department of Systems Biology, The University of Texas MD Anderson Cancer Center, Houston, TX 77030 USA; 5 Institute for Data Science in Oncology, The University of Texas MD Anderson Cancer Center, Houston, TX 77030 USA

**Keywords:** Transcriptional decomposition, Organogenesis, Cell differentiation, Oncofetal reprogramming, Stemness, ScRNA-seq, Pan-cancer, TCGA, Gene expression, Prognostic and predictive biomarkers

## Abstract

**Background:**

Cancer cells evolve under unique functional adaptations that unlock transcriptional programs embedded in adult stem and progenitor-like cells for progression, metastasis, and therapeutic resistance. However, it remains challenging to quantify the stemness-aware cell state of a tumor based on its gene expression profile.

**Methods:**

We develop a developmental-status-aware transcriptional decomposition strategy using single-cell RNA-sequencing-derived tissue-specific fetal and adult cell signatures as anchors. We apply our method to various biological contexts, including developing human organs, adult human tissues, experimentally induced differentiation cultures, and bulk human tumors, to benchmark its performance and to reveal novel biology of entangled developmental signaling in oncogenic processes.

**Results:**

Our strategy successfully captures complex dynamics in developmental tissue bulks, reveals remarkable cellular heterogeneity in adult tissues, and resolves the ambiguity of cell identities in in vitro transformations. Applying it to large patient cohorts of bulk RNA-seq, we identify clinically relevant cell-of-origin patterns and observe that decomposed fetal cell signals significantly increase in tumors versus normal tissues and metastases versus primary tumors. Across cancer types, the inferred fetal-state strength outperforms published stemness indices in predicting patient survival and confers substantially improved predictive power for therapeutic responses.

**Conclusions:**

Our study not only provides a general approach to quantifying developmental-status-aware cell states of bulk samples but also constructs an information-rich, biologically interpretable, cell-state panorama of human cancers, enabling diverse translational applications.

**Supplementary Information:**

The online version contains supplementary material available at 10.1186/s13073-024-01393-6.

## Background

Cancer is a highly convoluted ecosystem in which individual cells occupy distinct functional states that vary in a myriad of phenotypes, such as proliferation rate, metastatic capacity, and drug resistance [1]. A complete dissection of the cell state of a tumor is of great value as it presents cellular heterogeneity, elucidates oncogenic mechanisms, and predicts clinical outcomes. To make direct links to a specific phenotype, the tumor state is usually approximated by its gene expression profile, which can be further reduced to structured molecular subtypes, custom-defined gene signatures, or prior-knowledge-based signaling pathways. However, these “reductionism” driven approaches often fail to capture the complexity of cancer phenotypes because of their intrinsic limitations. With the advance of single-cell gene expression profiling, an attractive, holistic approach is to describe the state of a tumor with a set of orthogonal and highly diverse bases. Intuitively, these reference bases can be the discrete cell states that collectively constitute a tumor ecosystem. They would form a near-complete cell-state reference space where the projection coordinates of a tumor can be decomposed, and its related biological properties can be explained [2, 3].

Although resourceful in reshaping the gene expression program to maximize their fitness, cancer cells evolve new cell states generally under the constraint imposed by their original tissue signatures [4, 5]. The initiation and progression of cancer cells can be largely viewed as the reprogramming and dedifferentiation of adult tissue cells, which are characterized by a set of genomic, transcriptomic, and epigenomic features collectively termed “stemness” [6–10]. Although appealing in concept, it is hard to quantitatively characterize the “stemness” of a tumor in practice. Previous efforts involved extracting gene signatures from embryonic stem cells (ESC), induced pluripotent stem cells (iPSC), and in some cases, differentiated progenitors as well, and applying these gene signatures to quantify the stemness of a tumor sample [11–13], which show some power in the modeling of cancer cells’ aggressiveness. However, these studies have two major limitations. First, they largely ignored the tissue constraints and assumed the universality of a stemness gene signature across cancers originating from remarkably diverse tissue environments. Second, although it may be convenient to rely on the expression levels of stem cell-specific gene markers to directly “count” the abundance of their analogs in a tumor sample, the contamination of irrelevant cell types that also express these genes is almost inevitable. Thus, it is essential to account for the confounding effects of as many likely existing cell types as possible.

A meaningful cell state reference space where a tumor is anchored has to take into account the boundary set by tissue specificity and simultaneously be flexible on the time dimension, particularly the transition from the fetal phase to the adult phase in organ ontogeny. In this sense, recent studies [14–17] have demonstrated for individual contexts that tissue-specific fetal progenitor cells could be useful stemness “anchors,” as they yielded information-rich and biologically interpretable portrayals of tumor states. Great progress has been made in atlas-level gene expression profiling of mammalian tissue development at a single-cell resolution, usually detecting hundreds of different cell types [18–21]. Accordingly, a recent study [22] measured the all-by-all transcriptional similarities between human tumors and mouse fetal cell types, which yielded cell-of-origin insights in a pan-cancer manner. Expanding on these previous efforts, we aimed to construct a pan-tissue, species-aligned, context-specific, and full-developmental-status-aware digital cell-state reference, which serves as a basis for transcriptional decomposition [23, 24] to delineate the relative strength of cell programs in a highly heterogenous tissue ecosystem. We rigorously validated the utility of our approach in various biological scenarios and applied it comprehensively to large-scale patient cohorts of bulk RNA-seq data to unravel their tumor cell states. Our results demonstrate the incredible power of such an approach to characterizing the cell of origin, cancer stemness, and oncogenic activities, and conferring clinical utility such as modeling patient prognosis and drug resistance.

## Methods

### Data acquisition and processing

We obtained unique molecular identifier (UMI) counts of single cells from human and mouse tissues through the Human Cell Landscape (HCL: http://bis.zju.edu.cn/HCL/) [19] and Mouse Cell Atlas (MCA: http://bis.zju.edu.cn/MCA/) [18] data portals, respectively.

We obtained gene expression profiles (e.g., fragments per kilobase per million [FPKM] or transcripts per million [TPM]) of the TCGA cancer sample cohorts, the GTEx normal tissue cohorts, the MET500 metastatic tumor cohorts, and the CCLE cancer cell line cohorts, from the Xena data portal (https://xenabrowser.net/datapages/). We also obtained gene expression profiles of the human tissue development cohorts from ArrayExpress (https://www.ebi.ac.uk/arrayexpress/) under the accession ID E-MTAB-6814. We downloaded independent RNA-seq and microarray datasets of various biological contexts (raw or processed) from the Gene Expression Omnibus (GEO, https://www.ncbi.nlm.nih.gov/geo/) (Additional file 1: Table S1).

To convert gene-level FPKM values to TPM [25] values for a gene ${\text{g}}_{i}$ in a sample ${\text{s}}_{k}$, we used the formula:

$${{\textit{TPM}}_{{\mathit g}_{\mathit i}\mathit,{\mathit s}_{\mathit k}}}=\frac{{\textit{FPKM}}_{g_i,s_k}}{\sum_{j=1}^n{\textit{FPKM}}_{g_j,s_k}}\times10^6$$

where the denominator on the right side is the sum of the FPKM values of all the genes for an individual sample.

In re-processing RNA-seq data starting from fastq files, MultiQC [26] was used to assess the quality of the sequencing files and the performance of the preprocessing steps. Transcript-level abundances were quantified by Salmon [27] using the GRCh38 transcriptome as the reference. Gene-level TPM values were aggregated from transcript-level TPM values by tximport [28].

We downloaded the drug sensitivity data in cancer cell lines from the GDSC (https://www.cancerrxgene.org/) and the CTRPv2 (https://portals.broadinstitute.org/ctrp/) data portals. We used the area under the dose–response curve (AUC) as the metric to quantify drug resistance.

### Decomposition of bulk RNA-seq-based gene expression profiles

To computationally enumerate individual cell types from bulk RNA-seq samples, we used CIBERSORT [23] to estimate their relative fractions. Following the practices in the latest scRNA-seq-compatible update of CIBERSORT, CIBERSORTx [24], we constructed gene expression signature matrices (Additional file 2: Table S2) for the seven human tissues for which both fetal and adult samples were available in HCL, namely, adrenal gland, brain, kidney, liver, lung, pancreas, and stomach. Briefly, cells with < 500 UMI counts were defined as low-quality cells and removed for downstream analysis. The UMI counts were then normalized to CPM (count per million reads) to remove confounding factors introduced by variations in sequencing library size. Then, cells corresponding to the same human tissue, either fetal or adult, were combined and annotated according to the clustering results provided by the original studies, while maintaining the separation between fetal and adult tissues. Cell types supported by < 20 high-quality cells were removed to avoid bias in the subsequent decomposition analysis against those with extremely sparse signature expression vectors.

For each of the retained cell types, half of all the single cells were randomly selected without replacement and merged into a mega cell with average CPM values. Such random combinations were conducted ten times to generate replicates for each cell type and were used as an input for a differential gene expression (DGE) analysis to identify cell-type signature genes. Specifically, aggregated gene expression replicates of each cell type were compared against replicates of all the other cell types using a Mann–Whitney *U*-test, and the top 200 genes with an adjusted *p*-value of < 0.01 and the highest log_2_FC were defined as signature genes. The average expression levels of these genes across all single cells were pooled into a final signature matrix for that cell type. We excluded 671 genes involved in the cell cycle (GO:0007049), 108 genes involved in ribosome biogenesis (GO:0042254), 21 genes involved in cell apoptosis (GO:0008637), and 37 genes mapped to the mitochondrial genome. In the mouse-based decomposition, we identified one-to-one human-mouse ortholog pairs and followed the same procedure. With the signature matrices as a reference, we then decomposed the bulk RNA-seq samples into relative fractions of the corresponding tissue cell types.

### Collection of various molecular and clinical features of TCGA samples

We collected cancer drivers (including point mutations and somatic copy number variations) for each cancer type based on previous TCGA studies. We selected three to five driver events for each cohort according to their recurrence frequencies reported by the TCGA MC3 Working Group [29] (retrieved from the Xena data portal) across the tumors and used them to stratify the tumors for the comparison of decomposed cell state fractions between groups. We obtained tumor subtype, clinical stage, and patient prognosis information from the Xena data portal.

### Fetal-adult co-clustering analysis

To visualize the co-clustering of cell types based on the developmental stage, we applied the CP10K normalization to the raw counts on a single-cell level and then computed the cell-type-average expression profiles within each biological replicate. Next, log1p transformation was applied, and highly variable genes were selected using a dispersion-based method (the maximum and minimum average expression of a gene were set to be 3 and 0.0125, respectively, and the lower expression dispersion cutoff was set to 0.5). After that, the effects of the total count per cell and the percentage of mitochondrial gene count were regressed out, and the normalized and regressed counts were scaled so that the data had unit variance and zero mean (any values above 10 were clipped). Finally, the first 50 principal components were calculated, and the data dimension was reduced through a 2-D UMAP projection.

For the visualization of fetal-adult cell-type signature separation, a similar procedure was conducted, except that the input was the cell-type-and-replicate-aggregated signature matrices constructed as described above instead of the original gene expression matrices.

In the analysis of tumor sample enrichment in the nearest neighbors of fetal samples, we employed a similar data normalization process and calculated Euclidean distances in a 10-principal-component space where the linearity of transcriptome similarity was retained. For each defined number of nearest neighbors for the fetal samples (taking average gene expression profiles as a centroid), we then measured the proportion of tumor, NAT, and normal samples in that subset relative to their total numbers.

### Survival analysis

We employed a Cox proportional hazards model to evaluate the correlation between the fetalness index and patient survival time with or without correction for the age of diagnosis and tumor stage, the two most informative prognostic factors in the clinic. A hazard ratio > 1 denotes a favorable outcome for patients with a low index value and vice versa. Kaplan–Meier plots were used to visualize the difference in survival outcomes between the two equal-sized patient groups (split by the median value of the index).

### Enrichment analysis for cancer hallmark genes

We employed an information-theoretic framework [30] to reveal hallmark signatures across cancer types that were enriched in genes correlated with the fetalness index. First, we calculated Spearman’s rank correlation coefficients between gene expression level in TPM and the fetalness index across primary tumor samples, followed by rank-transformation and division into 15 equal bins. Then, the iPAGE algorithm calculated a mutual information (MI) value between the gene ranks and the hallmark signature memberships (i.e., the number of genes belonging to a module in each bin). We performed a random permutation test to estimate the significance of these MI values so that significantly informative hallmarks were identified with high MI values and low *p* values. A significant, positive MI value reflects the enrichment of a hallmark in genes positively correlated with the index and vice versa.

### Analysis of fetalness correlation with stem-cell gene marker expressions

Normal and cancer stem cell marker genes were first collected from various online and literature resources. We calculated Pearson correlation coefficients for the fetalness index and the stem cell marker expressions across samples for each cancer type, where we obtained developmental decomposition profiles. To test whether these associations are enriched on the positive side for the stemness markers, we also calculated the correlations between fetalness and other gene expressions as a baseline distribution. We tested the difference between the two distributions using a two-sided Mann–Whitney *U*-test.

### Analysis of cancer dependency

We obtained CERES scores of 18,119 genes across 769 cancer cell lines of 26 lineages from the DepMap data portal (https://depmap.org/portal/depmap/). A conservative mapping between TCGA cancer types and cancer cell line lineages was employed to allow tissue-specific analysis in the following ten cancer types: GBM, LGG, KIRC, KIRP, KICH, LIHC, LUAD, LUSC, PAAD, and STAD. A threshold of −0.5 was used to classify genes into essential and non-essential groups, as recommended by the DepMap study. In each cancer type, we then compared the proportion of essential genes in the genes positively or negatively correlated with the fetalness index using Fisher’s exact test.

## Results

### Capturing developmental cell state shifts in human tissues through single-cell decomposition

**Fig. 1:**
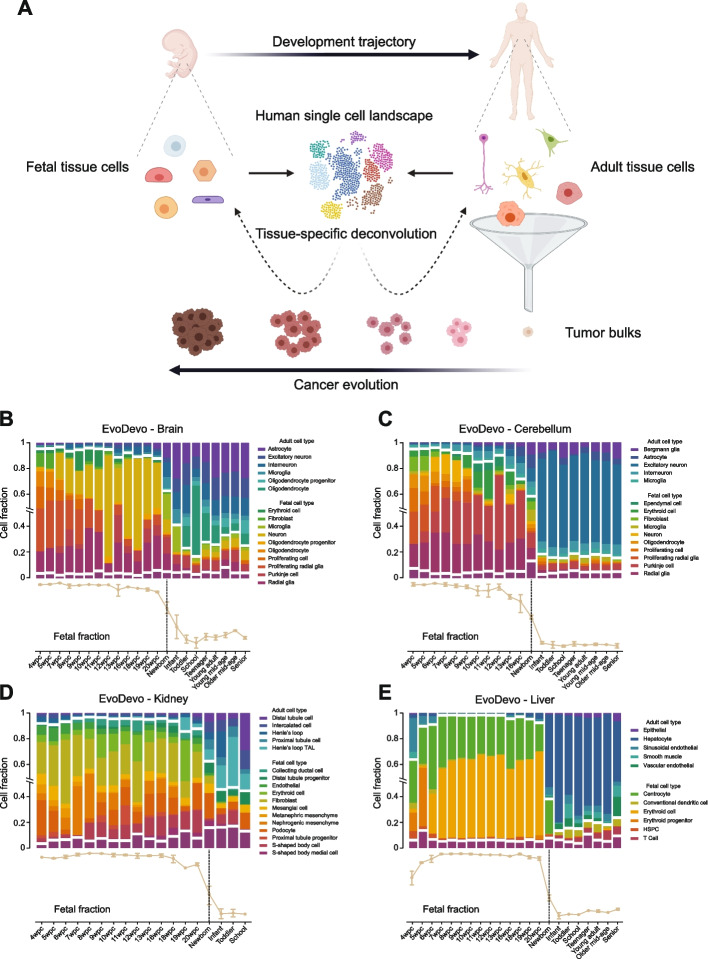
Overview and performance validation of a developmental-status-aware, tissue-specific decomposition strategy. **A** Schematic of the decomposition procedure. **B**–**E** Benchmarking of the decomposition approach using the EvoDevo tissue samples, including the human brain (**B**), cerebellum (**C**), kidney (**D**), and liver (**E**). Stacked bar plots in the top panels represent the relative fractions of cell types. An uncolored gap is added to separate the fetal and adult components. For each tissue, cell types with a maximum relative fraction of < 5% across all the samples are combined and shown as a single bar at the bottom, separated from others with an additional gap. Trendlines in the bottom panels show the changes in total fetalness along the developmental trajectory. Error bars denote 95% confidence intervals

To build a developmental-status-aware, tissue-specific cell-state reference for various human tissues, we compiled a series of single-cell RNA-seq data from both fetal and adult samples in the Human Cell Landscape (HCL) cohort [19]. We first identified significantly over-expressed genes in each of the annotated cell types and then used them to build a signature matrix, as described previously (Additional file 2: Table S2) [23, 24] (Fig. 1A). To reduce the bias that might confound the decomposition process, we excluded genes involved in the cell cycle, ribosome biogenesis, cell apoptosis, and mitochondrial genome. In this study, we focused on seven major human solid tissues where both fetal and adult single-cell data were available, namely the adrenal gland, brain, kidney, liver, lung, pancreas, and stomach. Through a UMAP [31] projection of the signature matrices, we found that fetal cell types were globally distinct from the adult cell types in each tissue, even in cases where a fetal cell type and its adult counterpart were essentially the same cell type (e.g., fetal and adult macrophages), indicating that the developmental status is a major determinant of cellular gene expression programs (Additional file 3: Fig. S1A–G). Importantly, the observed fetal-adult separation was not due to a sample-specific batch effect, as the HCL cell types from different biological replicates (donors) were clustered by the developmental stage rather than their sample origin (Fig. S1H). Finally, given a specific tissue, we decomposed bulk RNA-seq data based on the corresponding single-cell expression signatures using CIBERSORT [23, 24] to obtain the relative strength of individual cell state programs (Fig. 1A).

The ontogenesis of a human organ into its adult stage is characterized by an extensive turnover of a myriad of cell types, along with the transformation of gene expression programs within each cell type. We hypothesized that our decomposition strategy could effectively encapsulate such complex and dynamic cellular compositions from a series of bulk gene expression profiles. To test this hypothesis, we collected RNA-seq data from the EvoDevo study [32], where the complete developmental trajectories of the human brain, cerebellum, kidney, and liver tissues were well characterized. Based on this gold standard, we assessed whether the developmental dynamics could be accurately recovered. Indeed, our decomposition results (i) captured the global trend of a gradual loss of fetal cell types, (ii) identified the transition point exactly matching the newborn in each tissue, and (iii) provided detailed molecular portraits of specific cell types that are well-aligned with established findings (Fig. 1B–E). For example, in the brain tissue, there were barely any neurons at five weeks post-conception (wpc), the bulk being composed of neuronal progenitors such as the radial glia (Fig. 1B), consistent with the fact that neuronal production does not start until six wpc [33]. At seven wpc, the tissue swiftly transforms into a fetal-neuron-rich compartment, and after birth, it eventually becomes a fully functional ecosystem with a much higher cellular diversity where astrocytes, oligodendrocytes, neurons, glia cells, and progenitors co-exist (Fig. 1B). Similarly, in the liver, a transition from erythroid progenitors into erythroid cells occurs at seven wpc and erythroid cells are then maintained as the dominant cell type in the fetal liver until at least 20 wpc (Fig. 1E), corroborated by a recent time-course scRNA-seq study on the human fetal liver [34]. These results confirmed the accuracy of our decomposition approach in capturing the developmental stage of cellular composition transformation from bulk RNA-seq data.

### Revealing cellular heterogeneity of human adult tissues at a high resolution

**Fig. 2:**
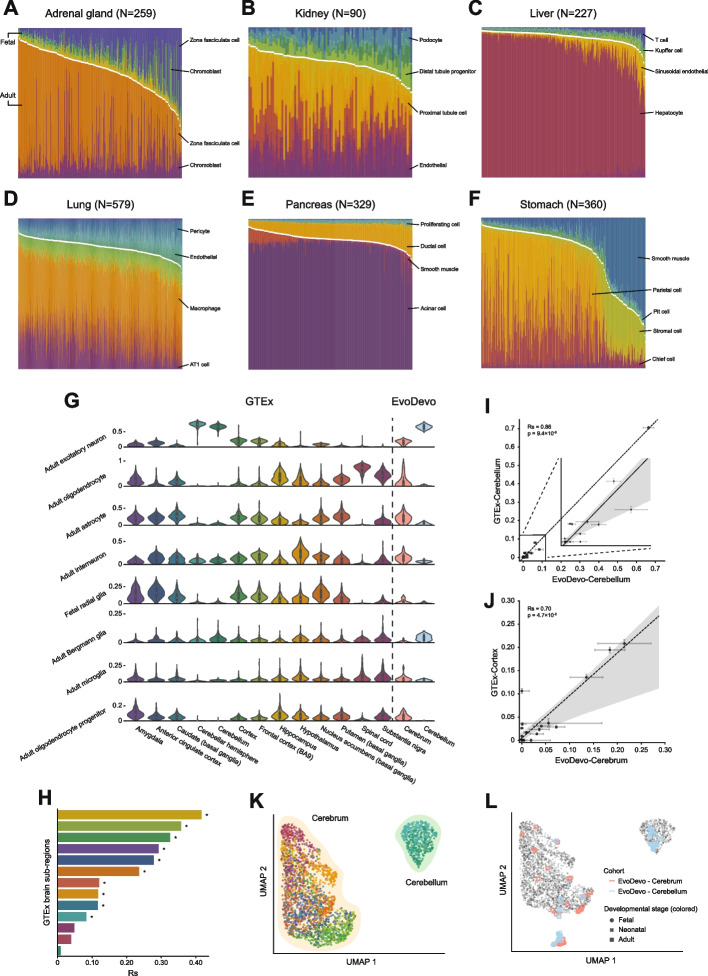
Cell-state decomposition of human adult normal tissues. **A**–**F** Stacked bar plots showing the fractions of cell types inferred in 6 GTEx tissues, i.e., adrenal gland (**A**), kidney (**B**), liver (**C**), lung (**D**), pancreas (**E**), and stomach (**F**). An uncolored gap is added to separate the fetal (top) and adult components (bottom). Samples are ordered by the total proportion of inferred tissue-specific fetal cell types, from the smallest to the largest. The top two fetal and adult cell types with the highest relative fractions are highlighted. **G** Violin plots showing the relative fractions of 8 brain cell types across the 13 GTEx brain sub-regions and two EvoDevo brain cohorts. **H** Bar plots showing Spearman’s rank correlation coefficients between total adult cell fractions and individual age across 13 GTEx brain sub-region cohorts. Statistically significant correlations (FDR < 0.05) are indicated with asterisks. **I** Correlation of estimated fractions of all 22 brain cell types between the GTEx cerebellar cohort (including the cerebellum and cerebellar hemisphere) and the EvoDevo cerebellum cohort. The translucent band around the regression line indicates the 95% confidence interval for the regression estimated through a bootstrap. **J** Same as **I** but for GTEx cerebral cohorts (including the cortex and frontal cortex [BA9]). **K** UMAP projection of GTEx brain sub-region samples based on inferred relative cell fractions. **L** UMAP projection in **K** overlaid with another UMAP projection of the EvoDevo brain samples. Grey circles indicate GTEx samples from **K**, which form the background to highlight EvoDevo samples

To demonstrate the capacity of our decomposition strategy to reveal the detailed cellular composition of human tissues, we decomposed > 4000 human samples of seven adult tissues from a cohort of a much larger scale, the Genotype-Tissue Expression (GTEx). We successfully characterized the cellular composition of these tissues, largely matching classical anatomic and physiological observations (Fig. 2A–G). For example, we found that the adrenal gland samples were mainly composed of zona fasciculata cells (Fig. 2A), which are known to occupy the middle and also the largest zone of the adrenal cortex [35]. The second most common cell type was the intra-adrenal chromoblast, which serves as the progenitor to the chromaffin cells [36] (not annotated by the HCL study) that take up most of the adrenal medulla [37]. Likewise, we inferred that the most common cell type (~ 80%) in the liver was hepatocytes (Fig. 2C), highly consistent with the anatomical estimation (80%) of this cell type in the tissue mass [38]. The second common cell type was the sinusoidal endothelial cell, which represents ~ 15% of the liver cells [39].

Notably, two observations seem to contradict the canonical views of adult tissue anatomy but can be well justified by recent literature. First, we detected fetal cell programs in all seven tissues, and in some cases, they composed a large proportion of the overall transcriptional state. For instance, fetal zona fasciculata cells constituted 12.4% (median) of the adrenal glands (Fig. 2A), which likely reflects the existence of a group of progenitor cells adopting a zona fasciculata phenotype, as reported in a study on a mouse model [40]. We found comparable cases, such as the presence of fetal distal tubule progenitors in the kidney (Fig. 2B), fetal pericytes in the lung (Fig. 2D), and fetal radial glia in the brain (Fig. 2G), all of which have been confirmed as sources for adult tissue maintenance and regeneration [41–43]. Thus, the presence of fetal cell types in the decomposition profiles suggests the intrinsic heterogeneity of human adult tissues and the inhabitation of stem or stem-like cells that naturally bear a resemblance to their fetal counterparts in their gene expression signature. Second, in contrast to the common view that the lung consists of mostly capillary endothelial cells and type I and type II pneumocytes [44], we estimated a considerable proportion of the lung mass to be resident macrophages (4.6–58.7%, median = 34.0%). Interestingly, a recent study using immune cell signatures to decompose samples across 46 GTEx tissues identified the lung as the organ with the highest macrophage infiltration [45]. Furthermore, a deeper, more comprehensive single-cell atlas study on the human lung revealed that macrophages were as abundant as 19.6–32.5% of the total lung mass [46]. This estimation is far above the classically determined abundance but very close to our decomposition.

To further assess the power of our decomposition strategy, we applied it to the GTEx brain cohort, where RNA-seq samples from 13 sub-regions were collected to allow a high-resolution, biologically informative investigation of cellular composition. We successfully recovered a series of signature cell types within each region, all supported by the literature. For instance, we noticed that although the brain sub-regions had variable cell compositions, the cerebral and cerebellar regions showed the sharpest contrast: the cerebellum was mostly enriched in excitatory neurons while largely depleted of oligodendrocytes that were prevalent in the cerebrum. Indeed, by directly counting the absolute numbers of various brain cells in mice, a recent study estimated that oligodendrocytes composed < 10% of the cerebellar mass and varied from 20 to 40% in other brain regions, but the neurons formed as much as 80% of the mouse cerebellum [47]. In another example, we detected a high abundance of interneurons in the hypothalamus, which is reportedly developmentally derived from interneurons in the alar plate [48]. Furthermore, we observed that Bergmann glia cells comprised a higher fraction of the cerebellum than other regions, well-aligned with the notion that they are “specialized astrocytes” in the cerebellum [49]. Finally, previous studies show that in the mouse brain, a few progenitor cell types declined during aging, although the global cellular composition remained largely unchanged [50–53]. Consistently, we detected moderate but significant positive associations between the total adult cell fraction and age in 10 of the 13 brain regions (FDR < 0.05, Fig. 2H).

To validate our decomposition results of the GTEx brain cohort more quantitatively, we decomposed a cohort of human brain RNA-seq profiles from the EvoDevo study [32]. We found that the estimations in the two independent cohorts were in full agreement both globally and locally, and the inferred cell compositions were very highly correlated (Fig. 2G, I, Rs = 0.86; Fig. 2L, Rs = 0.70). Intriguingly, while the adult brain samples were clustered together based on the cerebrum-cerebellum separation, the fetal samples almost exclusively formed a single tight cluster that was located closer to the adult cerebrum in the UMAP-projected space (Fig. 2L). This observation suggests that the prenatal brain, despite having committed to a cerebral or a cerebellar fate, maintains a shared gene expression program that undergoes dramatic divergence through postnatal development. Collectively, our decomposition approach demonstrated great power in revealing cellular heterogeneity from bulk RNA-seq data and further provided a high-resolution cellular composition map of human adult tissues in which transcriptionally fetal-like cell types appeared to be pervasive.

### Resolving ambiguous cellular identities during cell state transitions

**Fig. 3:**
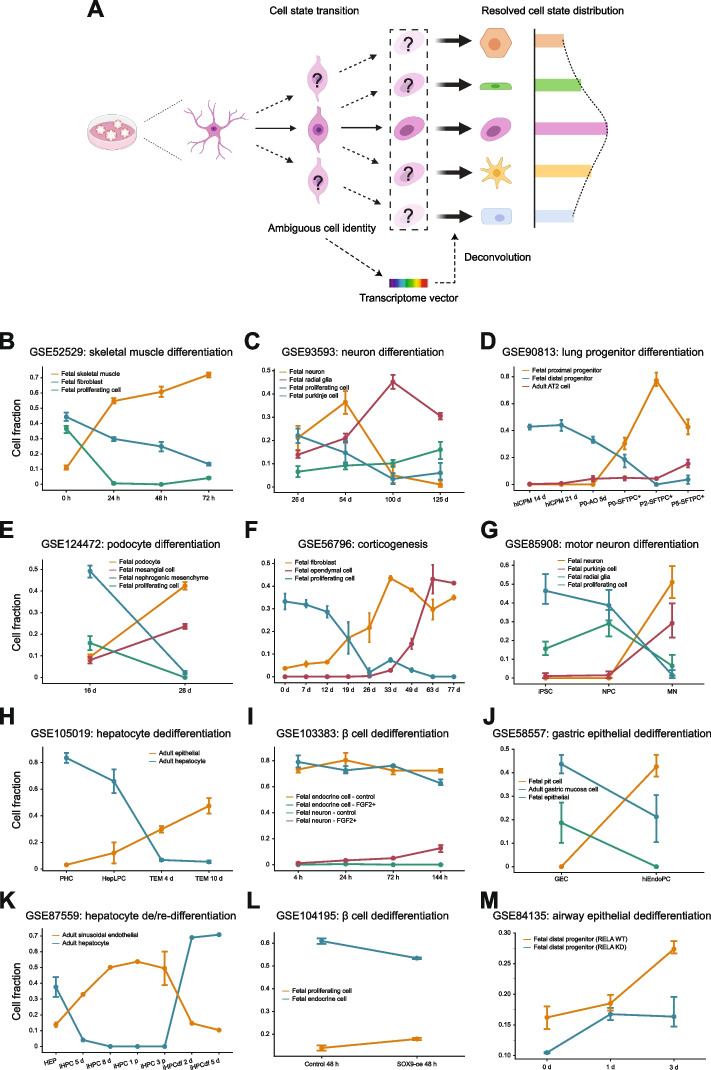
Decomposition-based cell state anchoring in in vitro transitions. **A** Schematic of the rationale for using decomposition to resolve the ambiguity of cell identities in an in vitro culture system based on their gene expression profiles. **B**–**M** Trendlines show alterations of relative cell fractions during cell state transformation in induced differentiation or de-differentiation assays. **D** CPM, carboxypeptidase M, a marker of ventralized anterior foregut endoderm (VAFE) cells; AO, alveolar organoids; P0/2/5, passages 0, 2, and 5; SFTPC, surfactant protein C, a marker of alveolar stem cells. **G** iPSC, induced pluripotent stem cell; NPC, neural progenitor cell; MN, motor neurons. **H** PHC, primary hepatocyte; HepLPC, liver progenitor-like cell; TEM, transition and expansion medium. **J** GEC, gastric epithelial cell; hiEndoPC, human-induced endodermal progenitor cell. **K** HEP, hepatocyte; iHPC, induced hepatic progenitor cell; iHPCdf, re-differentiated induced hepatic progenitor cell. Error bars denote 95% confidence intervals

In the above sections, our decomposition strategy showed an outstanding performance in quantifying the cell state composition of a bulk sample where the “ground truth” is known to some extent. We next aimed to assess the utility of this approach when a more flexible and dynamic definition of “cell state” is adopted, meaning that decomposition cell estimates may not have to reflect the heterogeneity of cell composition but represent the position of a mixture transcriptome (e.g., a tumor bulk) projected onto a cell state reference hyperspace where the developmental status is explicit. For this purpose, we applied our approach to in vitro cell culture systems under differentiation or de-differentiation, which bear two desirable properties. First, an in vitro cell culture is a mostly homogenous cellular community, and its cell state space is supposed to be constrained by the ground truth of the existent cell types. Second, such a system indeed experiences dramatic cell state transitions that are highly relevant to the tumor development process (Fig. 3A).

We compiled a series of in vitro time-course differentiation and de-differentiation RNA-seq profiles and applied our decomposition approach to them. We successfully recovered the major cell state programs in each culture system, along with the detailed alterations of their relative strength (skeletal muscle, neuron, AT2 cell, podocyte, ependymal cell, neuron, hepatocyte, β cell, gastric epithelial cell, hepatocyte, β cell, and distal progenitor cell in Fig. 3B–M, respectively). Moreover, our approach characterized the uncertainty of transient cell states by assigning the transcriptionally closest cell programs to the unknown/uncertain composition. For instance, in a case where lung AT2 cells were derived from iPSCs through lung progenitors [54], we observed the emergence of AT2 cell signals in the final stage of in vitro expansion, as originally reported. We were also able to clarify the identity of the intermediate cells as a mixture of the two major lung progenitor cell states, namely, the lung proximal and distal progenitors (Fig. 3G), with their shifts in strength perfectly reflecting their developmental succession [55]. In another example, when human pancreatic β cells were treated with FGF2 to induce de-differentiation into progenitors that did not have a ground-truth cell identity [56], we deduced that these developmentally upstream cells were of a fetal neuron identity (Fig. 3L). This trajectory has been suggested by previous studies on pancreatic development [57, 58]. Furthermore, across cases, we confirmed a strong resemblance of the cells at terminal differentiation points to fetal tissues rather than their mature counterparts (Fig. 3B–G), an intriguing phenomenon that has received extensive observations in diverse tissue contexts, including in brain [59–61], lung [62, 63], liver [64, 65], kidney [66, 67], and heart [68, 69]. Therefore, these results demonstrated that (i) our decomposition strategy was highly sensitive to the dynamics of lineage transformation and (ii) it can quantitatively resolve the uncertainty of cell states within a transiently heterogeneous phase, which is a hallmark of most physiological and disease processes, including the initiation and progression of cancer.

### Defining a developmental-status aware, cell state panorama of human cancers

**Fig. 4:**
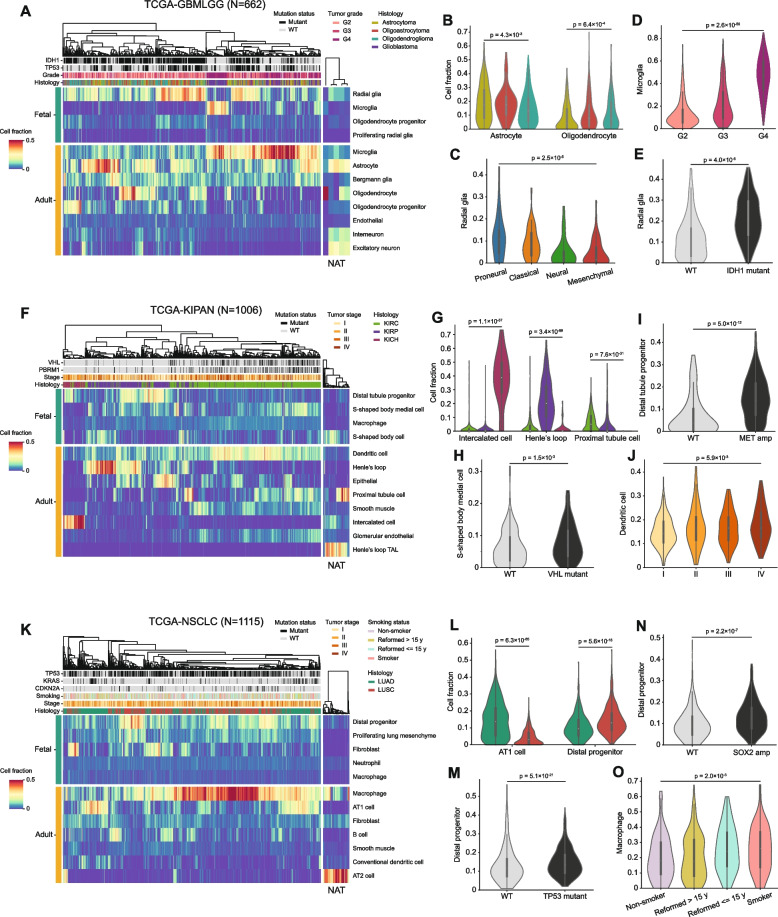
Cell state decomposition map of TCGA tumor samples. **A**, **F**, and **K** Heatmaps showing unsupervised clustering of inferred cell fraction profiles of tumors and adjacent normal samples in the pan-brain, GBM/LGG (**A**), pan-kidney, KIRC/KIRP/KICH (**F**), and pan-lung, LUAD/LUSC (**K**) cohorts. Feature tracks presented on the top of the heatmaps show mutation status, tumor grade/stage, histology types, and smoking status. **B**–**E** Violin plots showing differential cell fractions across three LGG histological subtypes (**B**), four established GBM molecular subtypes (**C**), three brain tumor grades (**D**), and LGG samples stratified by IDH1 mutation status (**E**). **G**–**J** Violin plots showing differential cell fractions across three kidney histological subtypes (**G**), KIRC samples stratified by VHL mutation status (**H**), KIRP samples stratified by MET amplification status (**I**), and four KIRC tumor stages (**J**). **L**–**O** Violin plots showing differential cell fractions across two lung histological subtypes (**L**), LUSC samples stratified by TP53 mutation status (**M**), LUAD samples stratified by SOX2 amplification status (**N**), and LUAD samples stratified by smoking status (**O**). A two-sided Mann–Whitney *U*-test or a one-way ANOVA (Kruskal–Wallis) test was used to calculate the *p*-value

Given the biologically meaningful cell-state transition results observed in in vitro experiments, we next applied our decomposition strategy to characterize the cell states of patient cancer samples from The Cancer Genome Atlas (TCGA) [4]. We found dramatic heterogeneity of adult and fetal-like cell states within and between different cancer types (Additional file 3: Fig. S2). Importantly, the decomposed cell state proportions showed significant correlations with well-annotated clinical and molecular features. In the brain tumor cohort (pan-glioma, Glioblastoma/Lower Grade Glioma, GBM/LGG), we discovered a significant depletion of adult neurons and an enrichment of multiple fetal cell types in the tumor samples compared to the normal adjacent to tumor (NAT) samples (Fig. 4A). Among the normally abundant adult cell types that also exist in tumor samples, the presence of astrocytes and oligodendrocytes significantly correlated with the corresponding tumor histology (Fig. 4B), indicating a good agreement with the known cells of origin. The GBM samples have been previously assigned to four molecular subtypes that largely reflect their cell states, namely, proneural, classical, neural, and mesenchymal [70, 71]. Consistent with the observation that proneural GBM tumors are enriched in neurogenesis processes, we found a significant over-representation of fetal radial glia cells, a major neuronal progenitor [72], in that subtype (Fig. 4C). Gliomas are known to be highly infiltrated by microglia/macrophages [73, 74]. Accordingly, our decomposition results not only confirmed the richness of microglia in GBM samples but also revealed an increasing trend of its content with disease progression (Fig. 4D). Additionally, we observed that fetal radial glia cells were significantly favorable in IDH1 mutant tumors (Fig. 4E), reminiscent of the role of this single most pervasive driver event in GBM/LGG in promoting an undifferentiated cell state [75].

In the kidney cancer cohort (pan-kidney, clear cell renal cell carcinoma/chromophobe renal cell carcinoma/papillary renal cell carcinoma, KIRC/KICH/KIRP), we observed a dramatic shift of cell identities between NAT and tumor samples as well as among tumors of different histological types (Fig. 4F). Assessing the cell origins of the three kidney cancer types, we found that KICH showed significantly higher similarity to normal adult intercalated cells, while KIRC was enriched in proximal tubule cells and glomerular endothelial cells (Fig. 4F, G), both consistent with previous studies [76, 77]. However, we detected a significant over-representation of Henle’s loop cells in KIRP, which deviated from the claim in the above-mentioned studies that KIRP and KIRC shared common cells of origin. Since these studies relied on gene expression correlations of tumor samples with normal bulk samples to infer the cells of origins, we suspected that the discrepancy could be due to improved resolution of our decomposition results using single-cell signatures. We then investigated the impacts of two major pan-kidney driver events [78, 79] on the cell state of the cancer types: VHL somatic mutations in KIRC contributed significantly to a cell state that favored S-shaped body medial cell (Fig. 4H), a known precursor for Henle’s loop and the distal tubule [80]; MET amplification was associated strongly with the accumulation of distal tubule progenitor cells in KIRP (Fig. 4I). Moreover, we detected a significant positive correlation between the tumor stage and the infiltration of dendritic cells in KIRC (Fig. 4J), suggesting a trend of increasing immunogenicity during the progression.

In the lung cancer cohort (pan-lung, lung adenocarcinoma/lung squamous carcinoma, LUAD/LUSC), in contrast to the observation that the NAT samples of the two diseases were uniformly enriched in AT2 cells, macrophages and fibroblasts, we uncovered a striking divergence of the cell states between LUAD and LUSC (Fig. 4K). Specifically, LUAD was characterized by a significantly higher fraction of type I alveolar cells (AT1), while LUSC was enriched in distal progenitor cells (Fig. 4L), a cell type known to be a multipotent progenitor population in the lung [81]. The abundance of such cells was positively correlated with TP53 mutations and SOX2 amplification in LUSC and LUAD, respectively (Fig. 4M, N). These results suggest a close relationship between early lung speciation and lung cancer initiation in terms of the cell of origin, as suggested in a recent study [82]. Interestingly, we observed extensive macrophage infiltration in the LUAD cohort associated with the patient’s smoking status (Fig. 4O), a connection independently reported before in normal lungs [83, 84]. Collectively, our decomposition strategy represents a powerful approach to understanding the cells of origins and cell states of clinical tumor samples.

### Quantifying cancer stemness based on a fetalness index

**Fig. 5:**
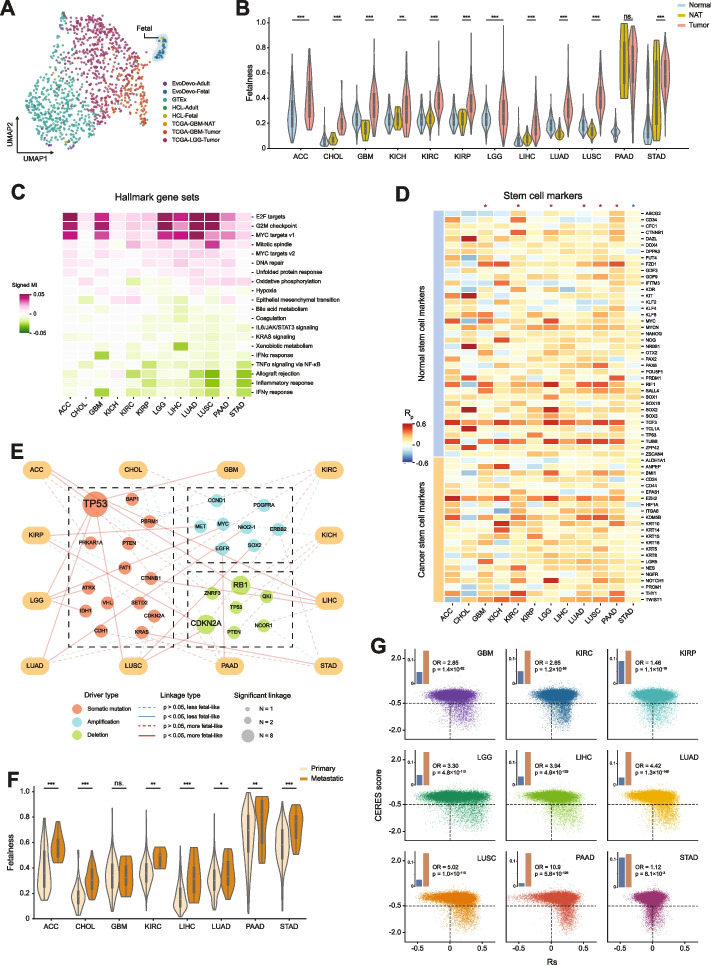
Molecular and clinical associations with the fetalness index across cancer types. **A** UMAP projection of normal brain and TCGA (GBM/LGG) samples based on estimated cell fractions with an exception for HCL samples where cell fractions were directly derived from single-cell profiling. Fetal samples are highlighted by the colored regions. **B** Violin plots showing a comparison of fetalness between tumor and NAT samples across 12 TCGA cancer types. When NAT samples are unavailable, the tumors are compared against GTEx normal samples. **C** Heatmap shows over- (pink) or under- (green) representation of hallmarks in genes positively correlated with the fetalness across primary tumor samples. **D** Heatmap showing Pearson correlation coefficients between the fetalness and the expression level of compiled normal and cancer stem-cell marker genes. Asterisks on top of the heatmap indicate that the correlation between the fetalness index and stem cell marker expressions is beyond (red) or below (blue) that between the fetalness index and the other genes. **E** Network depicting associations of a driver event (mutation or copy number variation) with either an increase or decrease of the fetalness in primary tumors. **F** Violin plots showing a comparison of fetalness between primary and metastatic tumors in 8 cancer types. **G** Scatter plots showing the relationship between cancer dependency and correlation with the fetalness of individual genes across nine cancer types. Bar plots represent the proportions of essential genes (a CERES score <  − 0.5) in genes either positively or negatively correlated with fetalness. A two-sided Mann–Whitney *U*-test (**B**, **D**, and **F**) or a Fisher’s exact test (**G**) was used to calculate *p* values. ns., non-significant, *FDR < 0.05, **FDR < 0.01, ***FDR < 0.001

The above analyses on individual cancers showed a consistent trend for tumor samples to be overall significantly enriched in tissue-specific fetal cell programs (Fig. 4A, F, and K). This observation suggested that through a process of reverse evolution, cancer cells yielded a more fetal-like cell state. To confirm this observation on a larger scale, we combined UMAP- projection-based visualization and PCA-based linear distance computing to explore the global topology of decomposed cell states of normal and tumor samples (TCGA/GTEx/EvoDevo), together with the original HCL single-cell reference set (see the “Methods” section). We found consistent proximity of tumor samples with either real fetal references or inferred fetal samples, compared to normal samples, across 12 cancer types (Fig. 5A and Additional file 3: Fig. S3A–S3G). Therefore, we reasoned that the aggregated fetal cell programs within a sample could be an informative index that can capture the evolutionary distance of a tumor cell state on a trajectory pointed towards a fetal-like state. Interestingly, when decomposing the TCGA samples using a mouse-based developmental cell-type reference, namely Mouse Cell Atlas (MCA) [18], we obtained similar results as in human-based decomposition (Additional file 3: Fig. S4A–S4I), suggesting a species-conserved adult-fetal transcriptome separation that mirrors cancer cell states. Using this index, we found that tumors across all the 12 cancer types had significantly higher fetalness than matched NAT samples and normal samples (Fig. 5B). We further confirmed these results based on non-TCGA cohorts of tumors and matched NAT samples (Additional file 3: Fig. S5A–S5L).

To investigate the biological relevance of the fetalness index, we performed a mutual information-based enrichment analysis [30]. We found that this index positively and significantly correlated with the over-representation of members of oncogenic hallmarks, including E2F targets, G2M checkpoints, and MYC targets. In contrast, the negatively correlated genes were mostly enriched for immune hallmarks, such as IFNγ response and inflammatory response (Fig. 5C). This index was also positively associated with the expression levels of many known normal and cancer stem-cell markers (Fig. 5D). As cancer progression is mainly modulated by driver genetic alterations that accumulate over time [85], we asked whether these driving events identified in multiple TCGA cancer types tend to correlate with an elevated fetal-like cell state by comparing tumors with and without top-ranked non-silent somatic mutations and copy number alterations. For nine of the 12 cancer types surveyed, we found at least one significant positive linkage with a more fetal-like cell state (Fig. 5E). The most common driver event that promoted an increase in the fetalness in many cancer types was TP53 mutations (Fig. 5E), consistent with its role in the stemness of human cancers [86, 87].

Metastasized tumors comprise cancer cells at an advanced evolutionary stage, bearing significantly more aggressive phenotypes than their primary counterparts. We hypothesized that the cell states of these tumors were more fetal-like and could be captured by our fetalness index. Using the index, we examined the metastatic samples from a large-scale multi-cancer-type cohort, MET500 [88], and found that metastatic tumors, indeed, were significantly more fetal-like in almost all the cancer types examined (Fig. 5F). These results indicated that the fetalness index, from a perspective of developmental resemblance, provides a high-level, meaningful summarization for the oncogenic dedifferentiation of primary and metastatic tumors.

Given the universal effect of our fetalness index in characterizing cancer aggressiveness, we next explored its utility in identifying genetic perturbations that could lead to a significant reversal of the cancer cell state. Recent large-scale genetic screening studies generated rich information on the survival dependency of cancer cells on nearly all protein-coding genes [89–91], providing a quantitative way for evaluating the “phenotypic importance” of a gene. Using a CRISPR-based screening dataset generated in cancer cell lines that histologically match nine TCGA cancer types in our analysis, we measured, in each cancer type, the favorability of being positively correlated with the fetalness index towards those that yielded significantly detrimental effect on cancer cells once knocked out (Methods). As expected, such correlation was strong among most cases, with a median odds ratio of 3.3, varying from 1.12 in STAD to 10.9 in PAAD (Fig. 5G). Collectively, using the developmental-status-aware, tissue-specific decomposition strategy, we uncovered extensive and meaningful molecular and phenotypic associations with a fetal-like cell state of human cancers.

Tumor bulk samples are known to be infiltrated or “contaminated” by non-cancer cells, and a series of computational approaches have been developed to quantify tumor purity [92–94]. Since our fetalness index relies on the tumor mixture transcriptome to assess its developmental resemblance, it is essential to investigate whether and how our index could be affected by such an intrinsic tumor characteristic. Across cancer types, correlation analysis revealed only moderate associations between the fetalness of a tumor and its stemness [12] or purity [92–94] measured in previous studies (Additional file 3: Fig. S6A). Although a positive correlation was present in some cases, we observed negative correlations in other cases as well (such as in ACC, KICH, and LIHC). We further compared the fetalness of different samples obtained from the same patients and observed a high concordance between samples (Rs = 0.67, *p* = 6.2 × 10^−4^, Additional file 3: Fig. S6B). These results suggested that the fetalness index, which quantifies a stem-like cell state, reflects an intrinsic feature of the tumor rather than certain technical factors, such as the variation in sample collection.

### The fetalness index shows robust prognostic power across cancer types

**Fig. 6:**
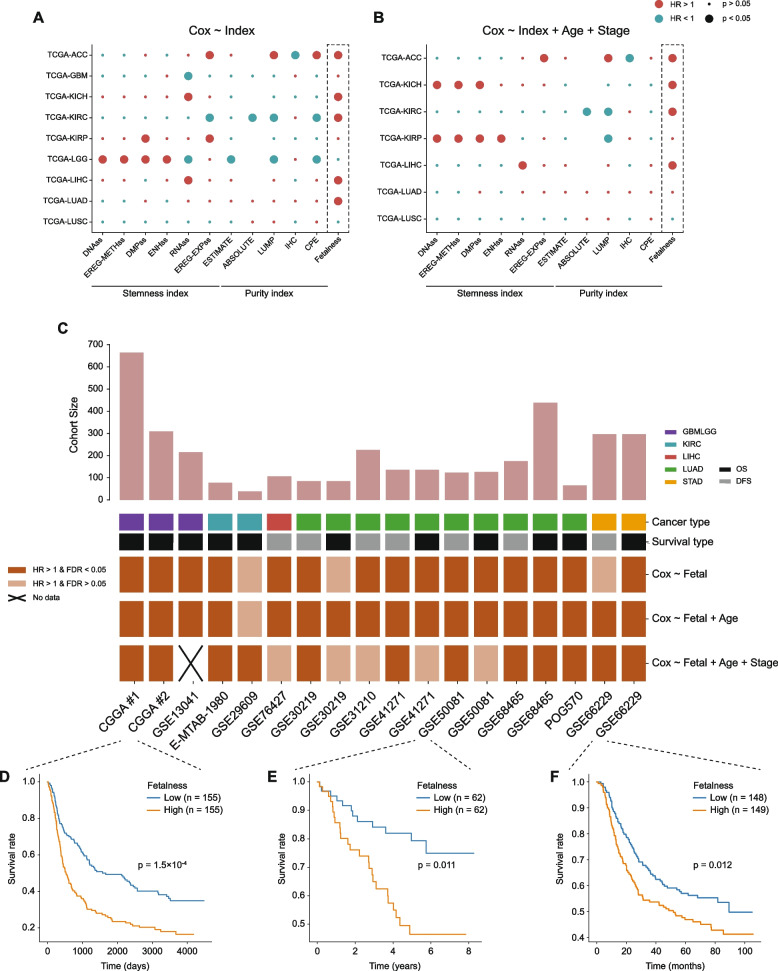
Correlations of fetalness with patient survival in multiple cancer types. **A**–**B** Survival analysis of TCGA cohorts using a univariate model that only includes the index of interest (**A**) or a multivariate model where the age of diagnosis and tumor stage are included (**B**). **C** Survival analysis of independent cancer patient cohorts, similar to **A**–**B**. The top-panel bar plots denote the cohort size, the middle-panel fetal tracks indicate cancer type and survival data type, and the bottom panel presents the statistical significance of each test. **D**–**F** Kaplan–Meier plots showing three representative examples where higher fetalness significantly correlates with worse patient survival

Next, we investigated the potential utility of our fetalness index as a prognostic signature in human cancers. Through a comparative analysis of its association with patient overall survival (OS), we demonstrated that this index showed a highly consistent and pervasive prognostic power across multiple cancer types (Fig. 6A and B). Importantly, regardless of whether evaluating with or without including other known prognostic factors (e.g., age and stage), this index showed higher robustness than a set of published purity and stemness indices across cancer types. For instance, although an expression-based stemness signature (RNAss) showed a significant prognostic power in four of the nine cancer types using a univariate Cox model, such predictions were in opposite directions for different cancer types and dramatically diminished when corrected for the age of diagnosis and disease stage.

To validate the findings in the TCGA cohorts independently, we collected a panel of gene expression profiles derived from patients with various cancer types where clinical and prognostic information were available. Among the 18 cohorts (5 cancer types) surveyed, we observed significant negative correlations between the fetalness and the probability of either overall survival or disease-free survival in 83.3% (15/18), 94.4% (17/18), and 76.4% (13/17) of the cases using a univariate Cox model, an age-included multivariate Cox model, or an age/stage-included multivariate Cox model, respectively (Fig. 6C–F). Furthermore, there were no cases of a statistically significant or non-significant positive correlation between the decomposed fetalness and survival probability in contrast to the published stemness indices, strongly supporting our index as a robust and consistent system-level prognostic signature for human cancers. Therefore, our fetalness index represents an independent, more robust prognostic marker in addition to clinical features.

### A fetal-like cell state predicts resistance to anti-cancer targeted therapies

**Fig. 7:**
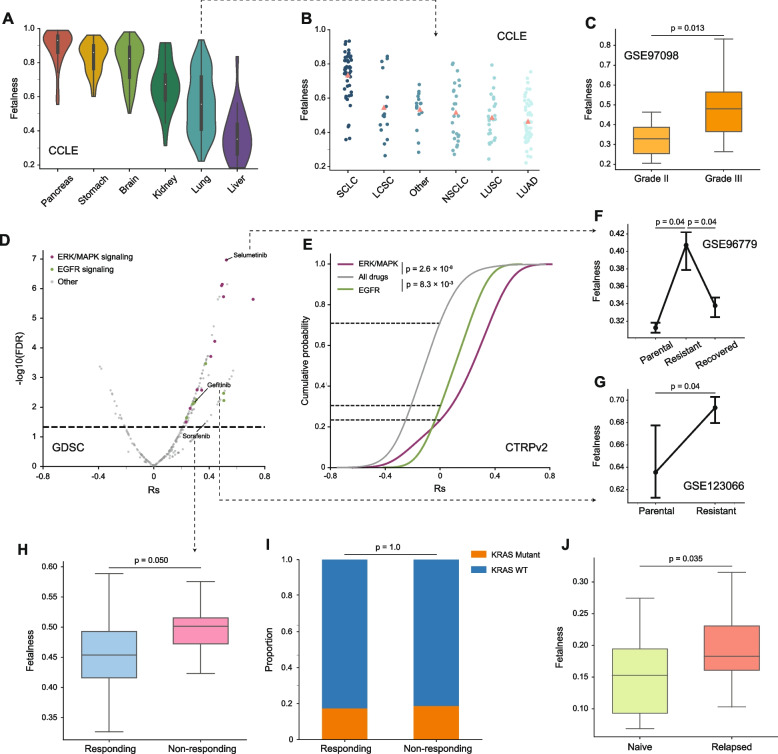
Correlations of the fetalness with resistance to various cancer therapies. **A** Violin plots showing the distributions of fetalness across cell lines of 6 lineages. **B** Strip plots showing the distributions of fetalness across cell lines of lung cancer subtypes. **C** Box plot showing the comparison of fetalness of liver cancer cell lines originating from grade II and III tumors. **D** Volcano plot showing the magnitude (*x*-axis) and statistical significance (*y*-axis) of cross-cell-line correlation between GDSC drug sensitivity (as measured by area under the dose–response curve) and fetalness. The horizontal dashed line indicates a significance threshold of FDR = 0.05. **E** Cumulative distributions of Spearman’s rank correlation coefficients between the fetalness and sensitivity to drugs targeting ERK/MAPK and EGFR pathways, or all drugs, in the CTRP dataset. **F**–**G** Effects of acquired drug resistance on fetal-like cell states under treatment of selumetinib (**F**) and gefitinib (**G**). **H** Box plot comparing the fetalness of pre-treatment patient tumor samples with differential responses to sorafenib treatment. **I** Bar plot showing the proportions of patients with and without KRAS mutation in responding and non-responding groups. **J** Box plot showing the comparison of the fetalness between the treatment naïve and the relapsed tumor patient groups. Box plots show the quartiles. The middle line indicates the median value; whiskers indicate the quartile ± 1.5 × interquartile range. A two-sided Mann–Whitney *U*-test or a Fisher’s exact test was used to calculate the p values except for **E**, where a Kolmogorov–Smirnov test was used. SCLC, small-cell lung cancer; LCSC, large-cell lung cancer; NSCLC, non-small-cell lung cancer; LUSC, lung squamous cell carcinoma; LUAD, lung adenocarcinoma

Oncogenic dedifferentiation, which leads to the accumulation of a pool of cancer stem cells, has long been considered a fundamental mechanism for tumors to acquire resistance to anti-cancer therapies [95, 96]. To demonstrate that our fetalness index quantifies the extent of cancer dedifferentiation to drug sensitivity, we performed an integrative analysis on a large panel of cell line gene expression profiles collected by the CCLE project [97, 98] and two independent drug sensitivity datasets, namely GDSC [99] and CTRPv2 [100]. Our decomposition strategy revealed great variations in the fetalness both within and across different lineages, which followed an order highly similar to that observed in primary tumor groups (Figs. 5B and 7A). Specifically, pancreatic and gastric cancer cell lines showed the highest fetalness, while liver cancer cell lines remained closer to their adult counterparts. We further examined the cohort of lung cancer cell lines in detail and found that small cell lung cancer (SCLC) cell lines had significantly higher fetalness (Fig. 7B), consistent with the observation that SCLC is the most aggressive subtype of lung cancer [101]. In a cohort of liver cancer cell lines where clinical grading was available [102], we observed a strong association between tumor grade and the fetal cell fraction (Fig. 7C). These results further supported that our decomposition strategy captured the dedifferentiation activity of in vitro cancer models as effectively as in patient samples.

We next focused on the CCLE lung cancer cell line cohort that had the largest overlap with the GDSC drug sensitivity dataset (*N* = 132) and assessed for each of the 266 anti-cancer drugs the association between the fetal-like state of a cell line (as the fetalness index) and the drug efficacy (as measured by the AUC of the dose–response curve) (Additional file 3: Fig. S7). Interestingly, we found a strong bias of GDSC drugs towards being ineffective in high-fetal-like cell lines (Fig. 7D), indicating a negative impact of oncogenic dedifferentiation on drug efficacy in general. We also noticed an enrichment of drugs targeting the ERK/MAPK and EGFR signaling pathways in the significant hits (FDR < 0.05, Fig. 7D), a pattern replicated in the independent CTRPv2 cohort (Fig. 7E). Given the strong correlations, we further asked if the acquired drug resistance in a single tumor model, as a common phenomenon in cancer treatment, was directly accompanied by the promotion of a highly fetal-like cell state. Indeed, we found that in cases where lung cancer cell lines were treated with increasing concentrations of two targeted inhibitors, selumetinib[103] and gefitinib (GSE123066), the cells exhibited a significantly increased fetal fraction (Fig. 7F and G). Furthermore, for selumetinib, there was a reversal into a low-fetal-like cell state once the treatment was stopped (Fig. 7F).

To assess the utility of the fetalness index in a more clinically relevant context, we examined its potential in monitoring NSCLC progression in patients in clinical trials. In a clinical trial for sorafenib [104], we found that poor responders had higher fetalness than those who showed clear clinical benefit, trending statistical significance (*p* = 0.050, FDR = 0.056) (Fig. 7H). Intriguingly, the mutation status of a major driver, KRAS, failed to predict the response to sorafenib (Fig. 7I) even though the drug was proposed to target KRAS-mutated tumors. This observation suggests that the presence of a major mutation per se, without clarifying its resultant cell-state transformation, is insufficient to predict the response to a targeted inhibitor. In another cohort where NSCLC patients were treated with various tyrosine kinase inhibitors [105], the fetalness showed a significant increase in relapsed tumors compared to the treatment-naïve ones (Fig. 7J). Collectively, these analyses indicate a close connection between the buildup of a fetal-like cancer cell state, through either positive selection for existing fetal cell states or adaptive shifts to new fetal programs, and the development of drug resistance. In terms of clinical utility, our results highlight the potential of the fetalness index as a biomarker for predicting/monitoring poor responses to targeted therapies.

## Discussion

Here, we proposed a decomposition strategy that benefited from (i) the inclusion of developmentally diverse cell state anchors and (ii) being constrained in a proper tissue-specific domain. We comprehensively validated its performance in diverse biological processes. First, using gene expression profiles encompassing well-annotated developmental trajectories of human tissues, our approach revealed a gradual and constant loss of fetal cell types, in conjunction with a detailed portrayal of the changes in cellular composition that match well-established physiological evidence, confirmed its accuracy in capturing highly dynamic developmental cell states. Second, when applied to a large cohort of human adult tissue samples, this approach also correctly recovered identities of major tissue cell types, as corroborated by the literature. Globally, we uncovered the pervasive presence of fetal cell programs in human adult tissues, consistent with the common knowledge that they maintain a repertoire of progenitor cells as a resource for self-renewal and regeneration under circumstances such as injury [106, 107]. In a detailed showcase of GTEx brain sub-regions, a global difference in cell composition between cerebellar and cerebral compartments was apparent and quantitatively reproducible based on independent brain cohorts. Furthermore, the inferred differences in cell composition across the sub-regions agreed well with multiple lines of anatomic evidence.

Our approach also helped resolve uncertainties in the distribution of cellular identity within a fast-changing transient cell community. Guided by a more generalized concept of cell state, our decomposition strategy generated detailed in vitro-induced differentiation and de-differentiation profiles that cover a wide range of tissue contexts. In addition to the validation of the transitions led by major and known cell types, we quantitatively determined the presence and identities of unknown and intermediate cell types. It should be emphasized that the estimated abundance of the cell identities, per our decomposition strategy, does not reflect our confidence in their existence. Rather, it establishes a transcriptional similarity linkage between unaccounted cell states and pre-defined tissue-specific cell signatures, thereby providing biologically meaningful insights into the functional and phenotypic state of an entire cellular ecosystem. Collectively, these results highlight the great capacity of our decomposition approach in depicting a high-resolution cellular heterogeneity map.

Similar to the situations in in vitro (de-)differentiation systems where cell states are indistinct and highly dynamic, cancer cells explore novel cell states to gain survival fitness through extensive transcriptional programming, largely deviating from their normal tissue counterparts. Thus, using our approach on bulk RNA-seq data from large patient cohorts, we have presented a developmental-status aware, cell state panorama of human cancers, both primary and metastatic tumors, which provides tremendous insights into the origin of cells, driver events, and cancer dependencies. Because the transcriptional decomposition process relying on linear decomposition is a similarity inference procedure in its essence, the decomposed proportions of cell types do not directly translate to their relative abundance in a tissue bulk. Rather, they are an estimation of the relative strength of different gene expression programs that may or may not be limited in a specific cell type when considered in its strict definition that goes beyond simply the transcriptional state. This is particularly true when the procedure is applied to detecting fetal cell signals from cancer tissues, as their physical connections and lineage relationships are far from well characterized. With these caveats, we believe that our development-aware decomposition approach yields meaningful results in placing cancer cell states in the context of organogenesis by quantitatively, albeit indirectly, measuring their similar biology.

Indeed, our study reveals that the total fetal cell program in the decomposition results is a meaningful index that can quantify the stemness of a tumor state. This index shows extensive correlations with well-established molecular and clinical features, including metastasis and stem cell markers. Notably, it only showed weak correlations with a set of published stemness indices based on the selected transcriptomic and epigenomic features from stem cells and progenitors [12]. Compared to these indices, our fetalness index showed a significantly improved prognostic power in terms of robustness and consistency. This is because of several reasons. First, in contrast to the previous studies, which lacked negative controls, namely features derived from differentiated adult cell types, we accounted for the presence of such components through an explicit separation of adult and fetal cell fractions. Second, we conducted cell-state anchoring in a tissue-specific manner, thereby ensuring a maximal alignment between the decoded cell state and the reference states. Last and probably the most important, cell state anchors were chosen to be fetal tissue cells rather than stem cells or progenitors of an earlier developmental phase where a tissue constraint had not been established. Besides prognostic power, through an interrogation of large-scale cell-line drug sensitivity data and independent patient cohorts from clinical trials, we demonstrated the power of our fetalness index in predicting response to anti-cancer therapies, offering a novel predictive marker and valuable insight into a mechanistic understanding of drug resistance. Currently, biomarkers are usually defined based on the mutation or expression status of individual genes or proteins. However, as we demonstrated, there are often no associations between such markers and clinical outcomes, even when the therapeutic targets are clearly defined. In contrast to biomarkers generated from such a reductionist approach, the decomposition approach represents a system-level characterization of how close a cancer cell state evolves to mirror fetal tissue programs. Thus, such cell states and their related indices would provide more effective guidance for developing precision cancer therapy.

Lastly, one puzzling observation from our analysis was the lack of a strong association between tumor fetalness and tumor purity. Because non-tumor cells normally do not acquire fetal characteristics, one would expect that the higher the tumor abundance, the higher the fetalness. While these associations are indeed mostly positive, we believe the reason they are moderate is that the sources of variation for tumor fetalness and tumor purity are independent of each other. Specifically, the former consists predominantly of oncogenic progression, while the latter is often caused by sampling bias during a surgical procedure (in the case of TCGA primary tumors). As demonstrated in our analysis, the fetalness of tumors within a single type, even those with very high tumor purity (e.g., LGG, > 80% purity for the vast majority), can span a wide spectrum. Thus, a situation is conceivable where even a smaller tumor proportion in a tissue bulk can exhibit very strong fetal characteristics and vice versa.

## Conclusions

In this study, we proposed a developmentally aware decomposition strategy that accurately identifies cell states across various biological contexts, from human developmental processes to adult tissues and cancers. By using tissue-specific cell state anchors and rigorous validation, we demonstrated its ability to detect subtle and dynamic cell transitions, including a pervasive fetal cell program in adult tissues. This approach also provided detailed insights into cellular composition, resolving uncertainties in transient cell communities and uncovering unknown intermediate cell types. In the cancer setting, our method revealed a developmental panorama of cancer states, correlating fetal cell programs with tumor progression and treatment responses. Importantly, we introduced a fetalness index that outperforms conventional stemness indices in predicting cancer prognosis and therapeutic outcomes, suggesting its utility as a novel biomarker for precision oncology. Collectively, these findings highlight the potential of our decomposition strategy in advancing our understanding of cellular heterogeneity and its implications for development, disease, and therapeutic intervention.

## Supplementary Information

Additional file 1: Table S1. Public bulk gene expression profiles for downstream decomposition analysis

Additional file 2: Table S2. Cell-type signature matrices of HCL adult and fetal tissues

Additional file 3: Fig. S1. Transcriptional separation of fetal and adult cell type references. Fig. S2. Deconvolved cell fractions of GTEx and TCGA samples. Fig. S3. Deconvolved cell fractions of GTEx and TCGA samples. Fig. S4. Correlation of fetalness estimated by HCL-based and MCA-based decomposition analyses. Fig. S5. Comparison of fetalness between NAT and tumor samples across non-TCGA cohorts. Fig. S6. Association of the fetalness index with tumor purity and published stemness indices. Fig. S7. Correlations of fetalness with drug response across GDSC cell lines

## Data Availability

Published gene expression profiling datasets are available in public data repositories such as the Gene Expression Omnibus (GEO). The descriptions and accession numbers for these datasets are listed in Additional file 1: Table S1. All original code has been deposited at Zenodo in the form of Jupyter Notebooks or Python scripts [108]. Any additional information required to reanalyze the data reported in this paper is available from the corresponding author upon request.
