# Supplementary material for: Developmental-status-aware transcriptional decomposition establishes a cell state panorama of human cancers
Source: Genome Med. 2024 Oct 28;16:124. doi: 10.1186/s13073-024-01393-6 (PMC11514945; doi:10.1186/s13073-024-01393-6)
Supplement: Supplementary file 3 — Additional file 3: Fig. S1. Transcriptional separation of fetal and adult cell type references. Fig. S2. Deconvolved cell fractions of GTEx and TCGA samples. Fig. S3. Deconvolved cell fractions of GTEx and TCGA samples. Fig. S4. Correlation of fetalness estimated by HCL-based and MCA-based decomposition analyses. Fig. S5. Comparison of fetalness between NAT and tumor samples across non-TCGA cohorts. Fig. S6. Association of the fetalness index with tumor purity and published stemness indices. Fig. S7. Correlations of fetalness with drug response across GDSC cell lines [file 13073_2024_1393_MOESM3_ESM.pdf]

Figure S1

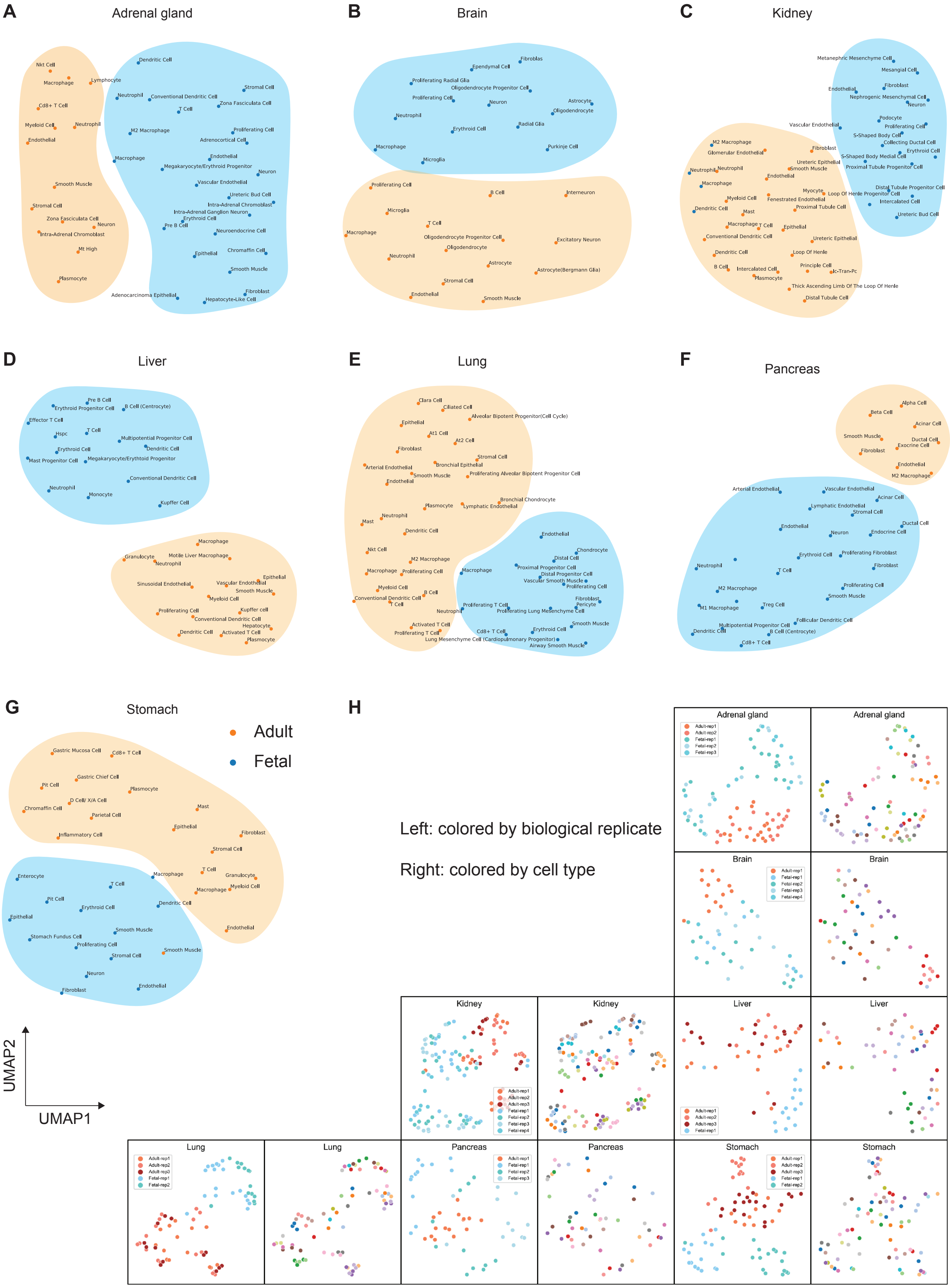
A
Adrenal gland
Nkt Cell
Macrophage
Lymphocyte
Cd8+ T Cell
Myeloid Cell
Neutrophil
Endothelial
Smooth Muscle
Stromal Cell
Zona Fasciculata Cell
Neuron
Intra-Adrenal Chromoblast
Mt High
Plasmocyte
Dendritic Cell
Neutrophil
Conventional Dendritic Cell
Stromal Cell
T Cell
Zona Fasciculata Cell
M2 Macrophage
Proliferating Cell
Adrenocortical Cell
Macrophage
Endothelial
Megakaryocyte/Erythroid Progenitor
Neuron
Vascular Endothelial
Ureteric Bud Cell
Intra-Adrenal Chromoblast
Intra-Adrenal Ganglion Neuron
Erythroid Cell
Pre B Cell
Neuroendocrine Cell
Epithelial
Chromaffin Cell
Smooth Muscle
Fibroblast
Adenocarcinoma Epithelial
Hepatocyte-Like Cell
B
Brain
Fibroblas
Ependymal Cell
Proliferating Radial Glia
Oligodendrocyte Progenitor Cell
Proliferating Cell
Neuron
Astrocyte
Oligodendrocyte
Neutrophil
Erythroid Cell
Radial Glia
Macrophage
Purkinje Cell
Microglia
Proliferating Cell
B Cell
Interneuron
Microglia
T Cell
Macrophage
Oligodendrocyte Progenitor Cell
Excitatory Neuron
Oligodendrocyte
Neutrophil
Astrocyte
Astrocyte(Bergmann Glia)
Stromal Cell
Endothelial
Smooth Muscle
C
Kidney
Metanephric Mesenchyme Cell
Mesangial Cell
Endothelial
Fibroblast
Nephrogenic Mesenchymal Cell
Neuron
Vascular Endothelial
Podocyte
Proliferating Cell
S-Shaped Body Cell
Collecting Ductal Cell
Erythroid Cell
S-Shaped Body Medial Cell
Proximal Tubule Progenitor Cell
Distal Tubule Progenitor Cell
Loop Of Henle Progenitor Cell
Intercalated Cell
Ureteric Bud Cell
M2 Macrophage
Glomerular Endothelial
Fibroblast
Ureteric Epithelial
Neutrophil
Neutrophil
Smooth Muscle
Endothelial
Macrophage
Myocyte
Myeloid Cell
Fenestrated Endothelial
Proximal Tubule Cell
Dendritic Cell
Mast
Macrophage
T Cell
Epithelial
Conventional Dendritic Cell
Ureteric Epithelial
Dendritic Cell
Loop Of Henle
B Cell
Intercalated Cell
Principle Cell
Ic-Tran-Pc
Plasmocyte
Thick Ascending Limb Of The Loop Of Henle
Distal Tubule Cell
D
Liver
Pre B Cell
Erythroid Progenitor Cell
B Cell (Centrocyte)
Effector T Cell
Hspc
T Cell
Multipotential Progenitor Cell
Erythroid Cell
Dendritic Cell
Mast Progenitor Cell
Megakaryocyte/Erythroid Progenitor
Conventional Dendritic Cell
Neutrophil
Monocyte
Kupffer Cell
Macrophage
Granulocyte
Motile Liver Macrophage
Neutrophil
Sinusoidal Endothelial
Vascular Endothelial
Epithelial
Smooth Muscle
Myeloid Cell
Proliferating Cell
Kupffer cell
Conventional Dendritic Cell
Hepatocyte
Dendritic Cell
Activated T Cell
Plasmocyte
E
Lung
Clara Cell
Ciliated Cell
Alveolar Bipotent Progenitor(Cell Cycle)
Epithelial
At1 Cell
At2 Cell
Fibroblast
Stromal Cell
Arterial Endothelial
Bronchial Epithelial
Smooth Muscle
Proliferating Alveolar Bipotent Progenitor Cell
Endothelial
Bronchial Chondrocyte
Plasmocyte
Lymphatic Endothelial
Neutrophil
Mast
Dendritic Cell
Endothelial
Nkt Cell
Chondrocyte
M2 Macrophage
Distal Cell
Macrophage
Proliferating Cell
Macrophage
Proximal Progenitor Cell
Distal Progenitor Cell
Vascular Smooth Muscle
Myeloid Cell
Proliferating Cell
B Cell
Conventional Dendritic Cell
T Cell
Fibroblast
Pericyte
Proliferating T Cell
Neutrophil
Proliferating Lung Mesenchyme Cell
Activated T Cell
Smooth Muscle
Cd8+ T Cell
Erythroid Cell
Proliferating T Cell
Lung Mesenchyme Cell (Cardiopulmonary Progenitor)
Airway Smooth Muscle
F
Pancreas
Alpha Cell
Beta Cell
Acinar Cell
Smooth Muscle
Ductal Cell
Exocrine Cell
Fibroblast
Endothelial
M2 Macrophage
Arterial Endothelial
Vascular Endothelial
Acinar Cell
Lymphatic Endothelial
Stromal Cell
Ductal Cell
Endothelial
Neuron
Endocrine Cell
Erythroid Cell
Proliferating Fibroblast
Neutrophil
Fibroblast
T Cell
M2 Macrophage
Proliferating Cell
Smooth Muscle
M1 Macrophage
Treg Cell
Multipotential Progenitor Cell
Follicular Dendritic Cell
Dendritic Cell
B Cell (Centrocyte)
Cd8+ T Cell
G
Stomach
Adult
Fetal
Gastric Mucosa Cell
Cd8+ T Cell
Gastric Chief Cell
Pit Cell
Plasmocyte
D Cell/ X/A Cell
Chromaffin Cell
Mast
Parietal Cell
Epithelial
Inflammatory Cell
Fibroblast
Stromal Cell
Enterocyte
Macrophage
T Cell
Granulocyte
T Cell
Macrophage
Myeloid Cell
Pit Cell
Dendritic Cell
Erythroid Cell
Epithelial
Smooth Muscle
Endothelial
Stomach Fundus Cell
Proliferating Cell
Smooth Muscle
Stromal Cell
Neuron
Fibroblast
Endothelial
UMAP2
UMAP1
H
Left: colored by biological replicate
Right: colored by cell type
Adrenal gland
Adrenal gland
Brain
Brain
Kidney
Kidney
Liver
Liver
Lung
Lung
Pancreas
Pancreas
Stomach
Stomach

Fig. S1. Transcriptional separation of fetal and adult cell type references

(A-G) UMAP projections of cell-type signatures based on normalized gene expressions (log-transformed CPM) across 7 tissues where both fetal and adult cells were collected by the HCL study, including adrenal gland (A), brain (B), kidney (C), liver (D), lung (E), pancreas (F), and stomach (G). Fetal and adult components are highlighted with colored regions. HCL, Human Cell Landscape.

(H) UMAP projections of HCL fetal and adult cell type average gene expression profiles, colored by HCL biological replicate (left) or cell type (right). Only the cell types overlapped between adult and fetal tissues were displayed.

Figure S2

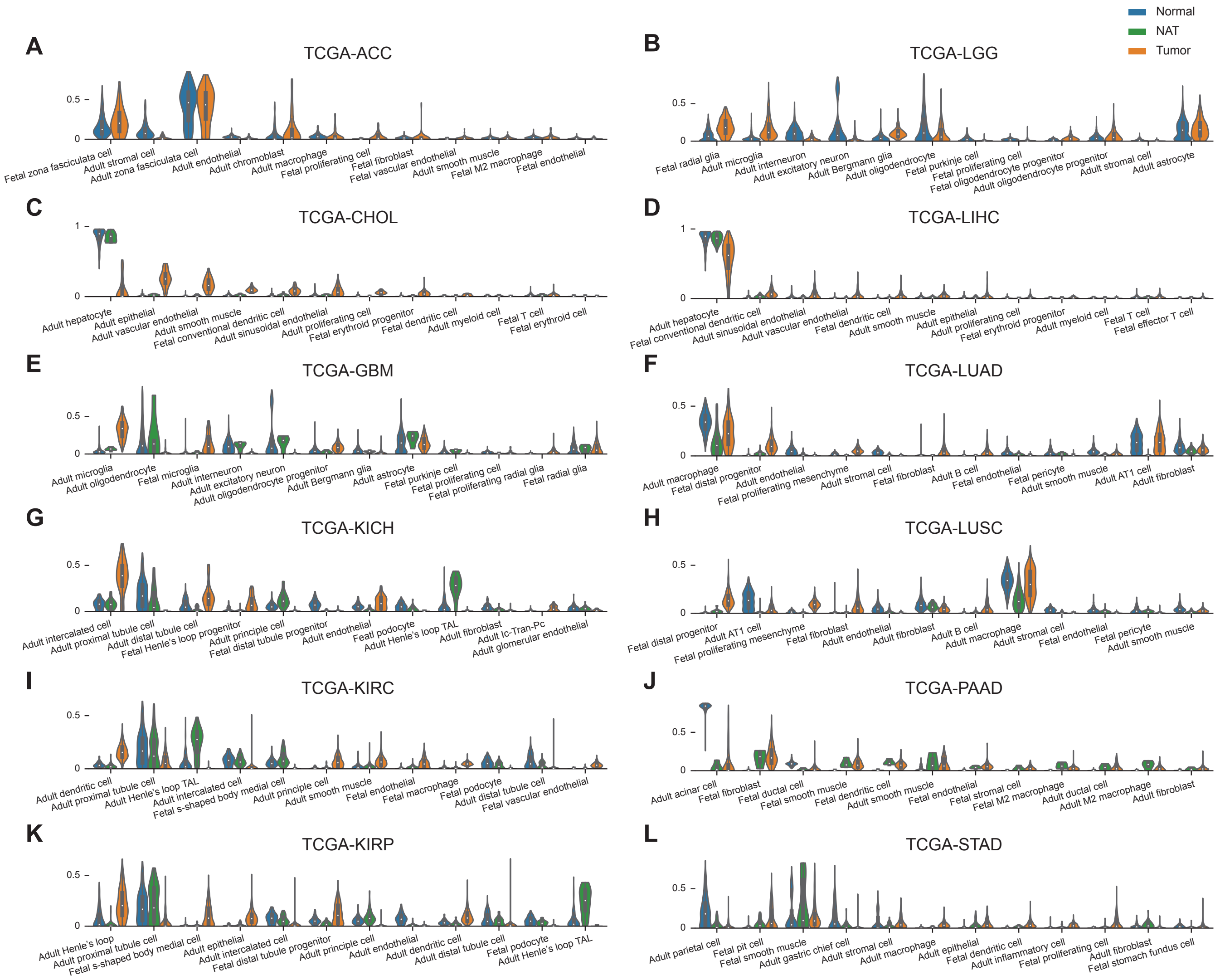
Normal
NAT
Tumor
A
TCGA-ACC
0.5
0
Fetal zona fasciculata cell
Adult stromal cell
Adult zona fasciculata cell
Adult endothelial
Adult chromoblast
Adult macrophage
Fetal proliferating cell
Fetal fibroblast
Fetal vascular endothelial
Adult smooth muscle
Fetal M2 macrophage
Fetal endothelial
B
TCGA-LGG
0.5
0
Fetal radial glia
Adult microglia
Adult interneuron
Adult excitatory neuron
Adult Bergmann glia
Adult oligodendrocyte
Fetal purkinje cell
Fetal proliferating cell
Fetal oligodendrocyte progenitor
Adult oligodendrocyte progenitor
Adult stromal cell
Adult astrocyte
C
TCGA-CHOL
1
0
Adult hepatocyte
Adult epithelial
Adult vascular endothelial
Adult smooth muscle
Fetal conventional dendritic cell
Adult sinusoidal endothelial
Adult proliferating cell
Fetal erythroid progenitor
Fetal dendritic cell
Adult myeloid cell
Fetal T cell
Fetal erythroid cell
D
TCGA-LIHC
1
0
Adult hepatocyte
Fetal conventional dendritic cell
Adult sinusoidal endothelial
Adult vascular endothelial
Fetal dendritic cell
Adult smooth muscle
Adult epithelial
Adult proliferating cell
Fetal erythroid progenitor
Adult myeloid cell
Fetal T cell
Fetal effector T cell
E
TCGA-GBM
0.5
0
Adult microglia
Adult oligodendrocyte
Fetal microglia
Adult interneuron
Adult excitatory neuron
Adult oligodendrocyte progenitor
Adult Bergmann glia
Adult astrocyte
Fetal purkinje cell
Fetal proliferating cell
Fetal proliferating radial glia
Fetal radial glia
F
TCGA-LUAD
0.5
0
Adult macrophage
Fetal distal progenitor
Adult endothelial
Fetal proliferating mesenchyme
Adult stromal cell
Fetal fibroblast
Adult B cell
Fetal endothelial
Fetal pericyte
Adult smooth muscle
Adult AT1 cell
Adult fibroblast
G
TCGA-KICH
0.5
0
Adult intercalated cell
Adult proximal tubule cell
Adult distal tubule cell
Fetal Henle's loop progenitor
Adult principle cell
Fetal distal tubule progenitor
Adult endothelial
Featl podocyte
Adult Henle's loop TAL
Adult fibroblast
Adult Ic-Tran-Pc
Adult glomerular endothelial
H
TCGA-LUSC
0.5
0
Fetal distal progenitor
Adult AT1 cell
Fetal proliferating mesenchyme
Fetal fibroblast
Adult endothelial
Adult fibroblast
Adult B cell
Adult macrophage
Adult stromal cell
Fetal endothelial
Fetal pericyte
Adult smooth muscle
I
TCGA-KIRC
0.5
0
Adult dendritic cell
Adult proximal tubule cell
Adult Henle's loop TAL
Adult intercalated cell
Fetal s-shaped body medial cell
Adult principle cell
Adult smooth muscle
Fetal endothelial
Fetal macrophage
Fetal podocyte
Adult distal tubule cell
Fetal vascular endothelial
J
TCGA-PAAD
0.5
0
Adult acinar cell
Fetal fibroblast
Fetal ductal cell
Fetal smooth muscle
Fetal dendritic cell
Adult smooth muscle
Fetal endothelial
Fetal stromal cell
Fetal M2 macrophage
Adult ductal cell
Adult M2 macrophage
Adult fibroblast
K
TCGA-KIRP
0.5
0
Adult Henle's loop
Adult proximal tubule cell
Fetal s-shaped body medial cell
Adult epithelial
Adult intercalated cell
Fetal distal tubule progenitor
Adult principle cell
Adult endothelial
Adult dendritic cell
Adult distal tubule cell
Fetal podocyte
Adult Henle's loop TAL
L
TCGA-STAD
0.5
0
Adult parietal cell
Fetal pit cell
Fetal smooth muscle
Adult gastric chief cell
Adult stromal cell
Adult macrophage
Adult epithelial
Fetal dendritic cell
Adult inflammatory cell
Fetal proliferating cell
Adult fibroblast
Fetal stomach fundus cell

Fig. S2. Deconvolved cell fractions of GTEx and TCGA samples

(A-L) Violin plots showing the estimated relative fractions of cell types within normal, NAT, and tumor samples across 12 TCGA cancer types, including TCGA-ACC (A), TCGA-LGG (B), TCGA-CHOL (C), TCGA-LIHC (D), TCGA-GBM (E), TCGA-LUAD (F), TCGA-KICH (G), TCGA-LUSC (H), TCGA-KIRC (I), TCGA-PAAD (J), TCGA-KIRP (K), and TCGA-STAD (L).

Figure S3

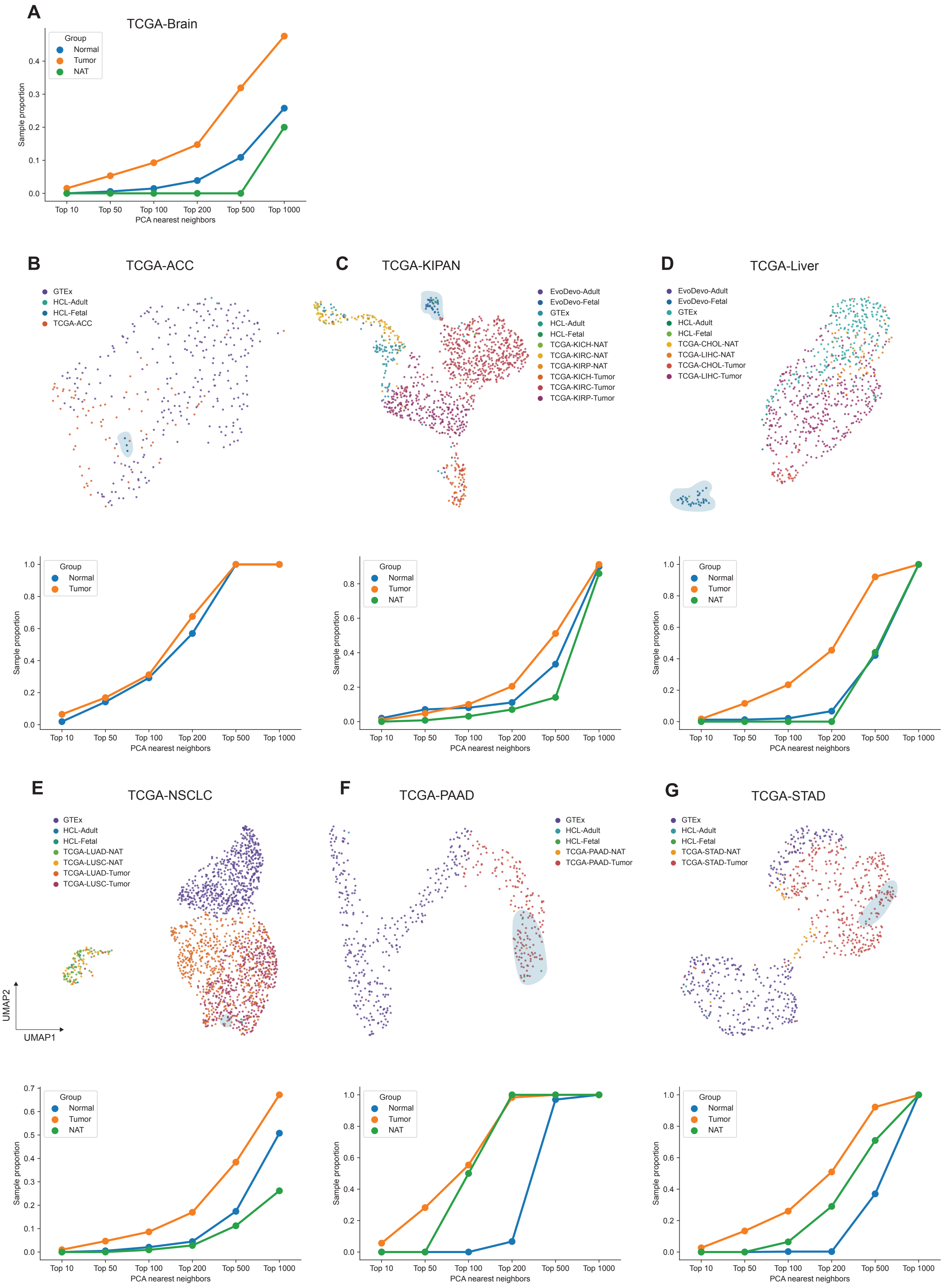

A
TCGA-Brain
Group
Normal
Tumor
NAT
Sample proportion
0.0
0.1
0.2
0.3
0.4
Top 10
Top 50
Top 100
Top 200
Top 500
Top 1000
PCA nearest neighbors
B
TCGA-ACC
GTEx
HCL-Adult
HCL-Fetal
TCGA-ACC
C
TCGA-KIPAN
EvoDevo-Adult
EvoDevo-Fetal
GTEx
HCL-Adult
HCL-Fetal
TCGA-KICH-NAT
TCGA-KIRC-NAT
TCGA-KIRP-NAT
TCGA-KICH-Tumor
TCGA-KIRC-Tumor
TCGA-KIRP-Tumor
D
TCGA-Liver
EvoDevo-Adult
EvoDevo-Fetal
GTEx
HCL-Adult
HCL-Fetal
TCGA-CHOL-NAT
TCGA-LIHC-NAT
TCGA-CHOL-Tumor
TCGA-LIHC-Tumor
0.2
0.4
0.6
0.8
1.0
E
TCGA-NSCLC
GTEx
HCL-Adult
HCL-Fetal
TCGA-LUAD-NAT
TCGA-LUSC-NAT
TCGA-LUAD-Tumor
TCGA-LUSC-Tumor
UMAP2
UMAP1
F
TCGA-PAAD
GTEx
HCL-Adult
HCL-Fetal
TCGA-PAAD-NAT
TCGA-PAAD-Tumor
G
TCGA-STAD
GTEx
HCL-Adult
HCL-Fetal
TCGA-STAD-NAT
TCGA-STAD-Tumor
0.5
0.6
0.7

Fig. S3. Deconvolved cell fractions of GTEx and TCGA samples

(A) Line plot showing the proportion of normal, tumor, or NAT samples in the nearest neighbors of fetal samples for the brain tissue context. (B-G) UMAP projection of sample-level cell composition profiles (top panel) or same as (A) (bottom panel) in tissue contexts matching TCGA-ACC (B), TCGA-KIPAN (C), TCGA-Liver (D), TCGA-NSCLC (E), TCGA-PAAD (F), and TCGA-STAD (G), based on estimated cell fractions with an exception for HCL samples where cell fractions are directly derived from single-cell profiling. In UMAP plots, fetal samples are highlighted by the colored region. NAT, normal adjucent tissue. HCL, Human Cell Landscape.

Figure S4

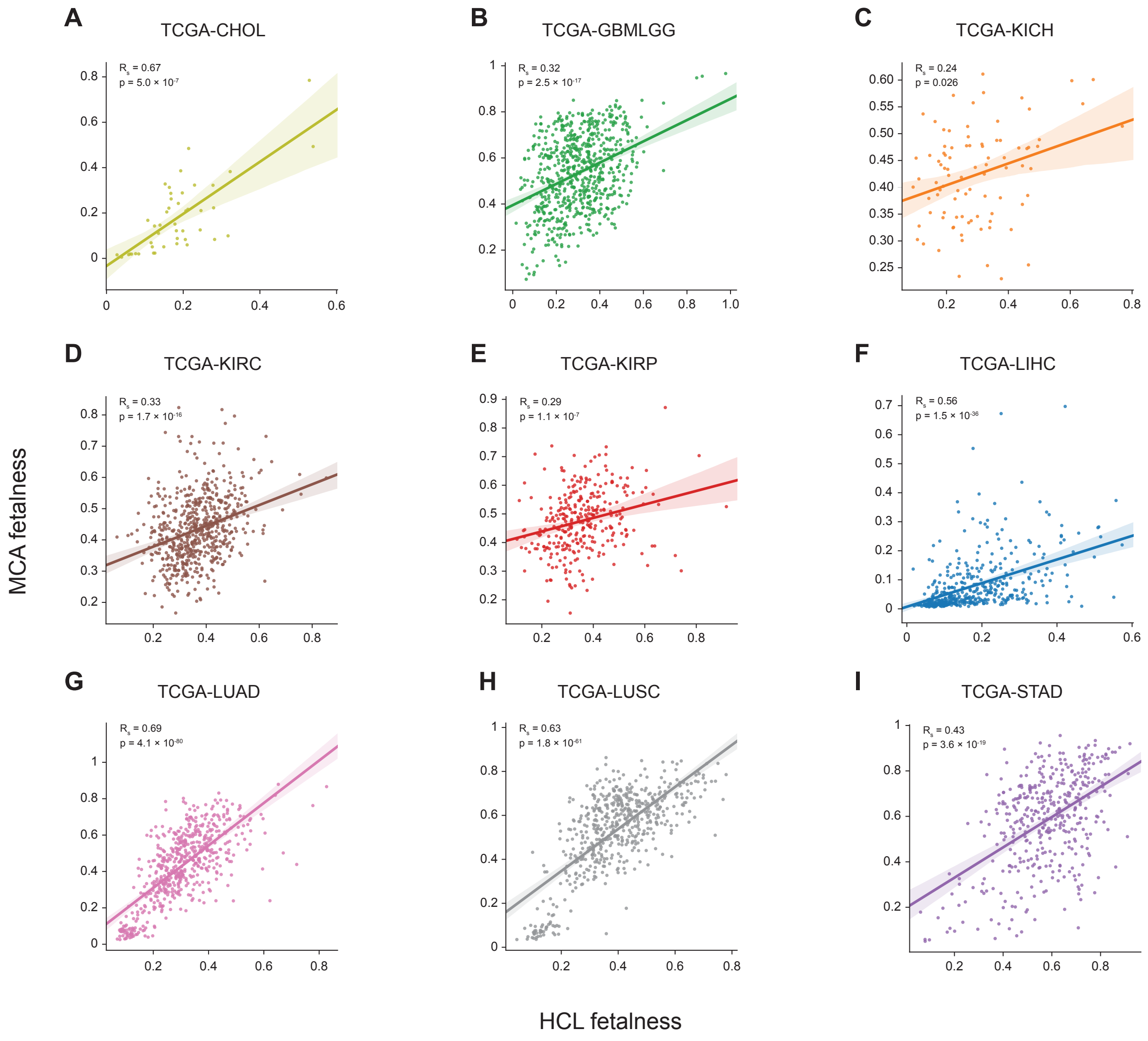
A
TCGA-CHOL
R_s = 0.67
p = 5.0 × 10^-7
B
TCGA-GBMLGG
R_s = 0.32
p = 2.5 × 10^-17
C
TCGA-KICH
R_s = 0.24
p = 0.026
D
TCGA-KIRC
R_s = 0.33
p = 1.7 × 10^-16
E
TCGA-KIRP
R_s = 0.29
p = 1.1 × 10^-7
F
TCGA-LIHC
R_s = 0.56
p = 1.5 × 10^-36
G
TCGA-LUAD
R_s = 0.69
p = 4.1 × 10^-80
H
TCGA-LUSC
R_s = 0.63
p = 1.8 × 10^-61
I
TCGA-STAD
R_s = 0.43
p = 3.6 × 10^-19
MCA fetalness
HCL fetalness

Fig. S4. Correlation of fetalness estimated by HCL-based and MCA-based deconvolution analyses

(A-I) Scatter plots depicting the relationship between HCL-deconvoluted and MCA-deconvolved fetalness across samples of 9 TCGA cancer types, including TCGA-CHOL (A), TCGA-GBM/LGG (B), TCGA-KICH (C), TCGA-KIRC (D), TCGA-KIRP (E), TCGA-LIHC (F), TCGA-LUAD (G), TCGA-LUSC (H), and TCGA-STAD (I). The colored regions around the regression lines indicate a 95% confidence interval. HCL, Human Cell Landscape; MCA, Mouse Cell Atlas.

Figure S5

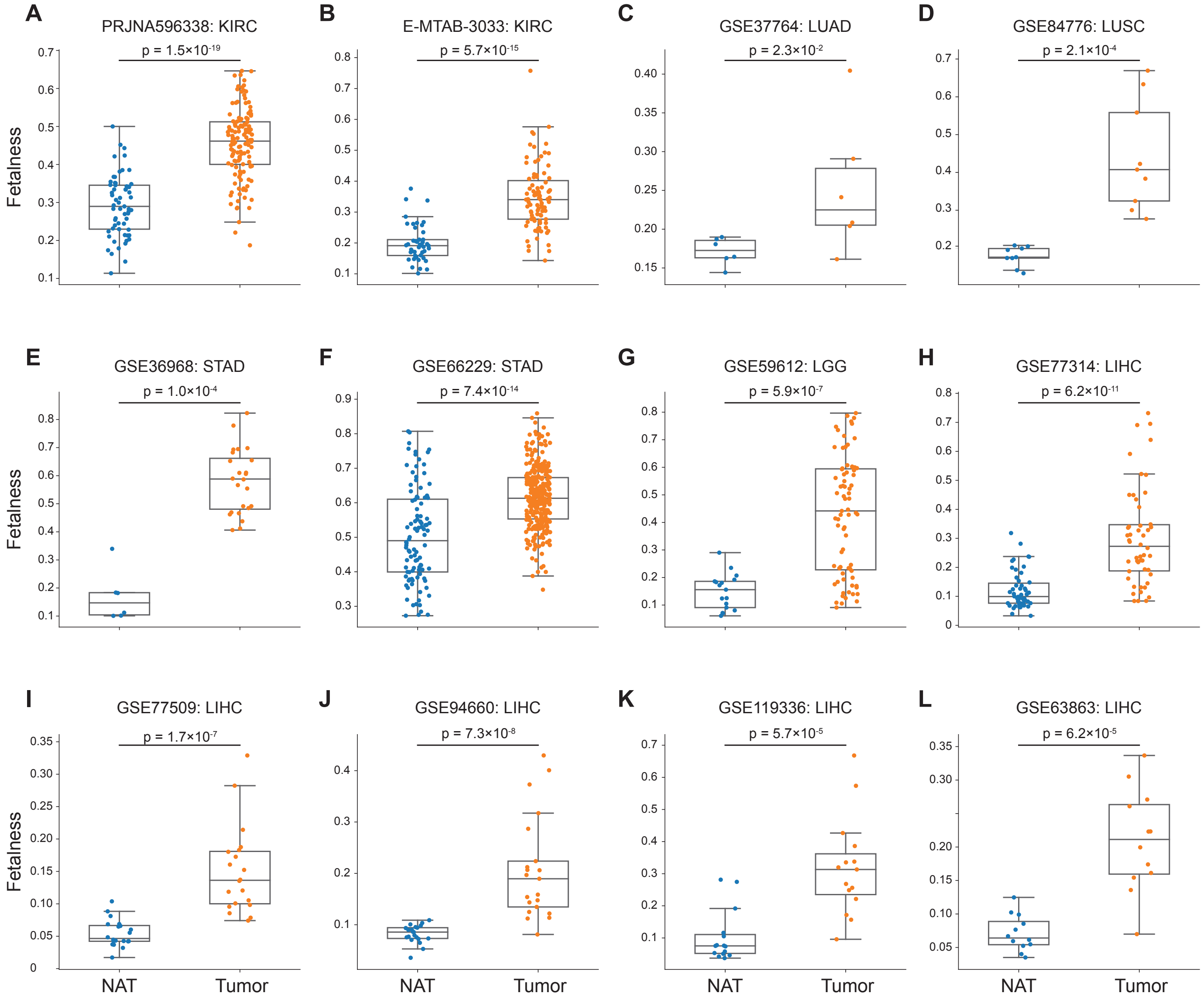

A
PRJNA596338: KIRC
p = 1.5×10-19
B
E-MTAB-3033: KIRC
p = 5.7×10-15
C
GSE37764: LUAD
p = 2.3×10-2
D
GSE84776: LUSC
p = 2.1×10-4
E
GSE36968: STAD
p = 1.0×10-4
F
GSE66229: STAD
p = 7.4×10-14
G
GSE59612: LGG
p = 5.9×10-7
H
GSE77314: LIHC
p = 6.2×10-11
I
GSE77509: LIHC
p = 1.7×10-7
J
GSE94660: LIHC
p = 7.3×10-8
K
GSE119336: LIHC
p = 5.7×10-5
L
GSE63863: LIHC
p = 6.2×10-5
Fetalness
NAT
Tumor

Fig. S5. Comparison of fetalness between NAT and tumor samples across non-TCGA cohorts

(A-L) Box plots showing the difference of fetal fractions between NAT and tumor samples in 12 non-TCGA patient cohorts. The box plots show the quartiles. The whiskers indicate the quartile ± 1.5× interquartile range. A two-sided Mann-Whitney U-test was used to calculate the p-value.

Figure S6

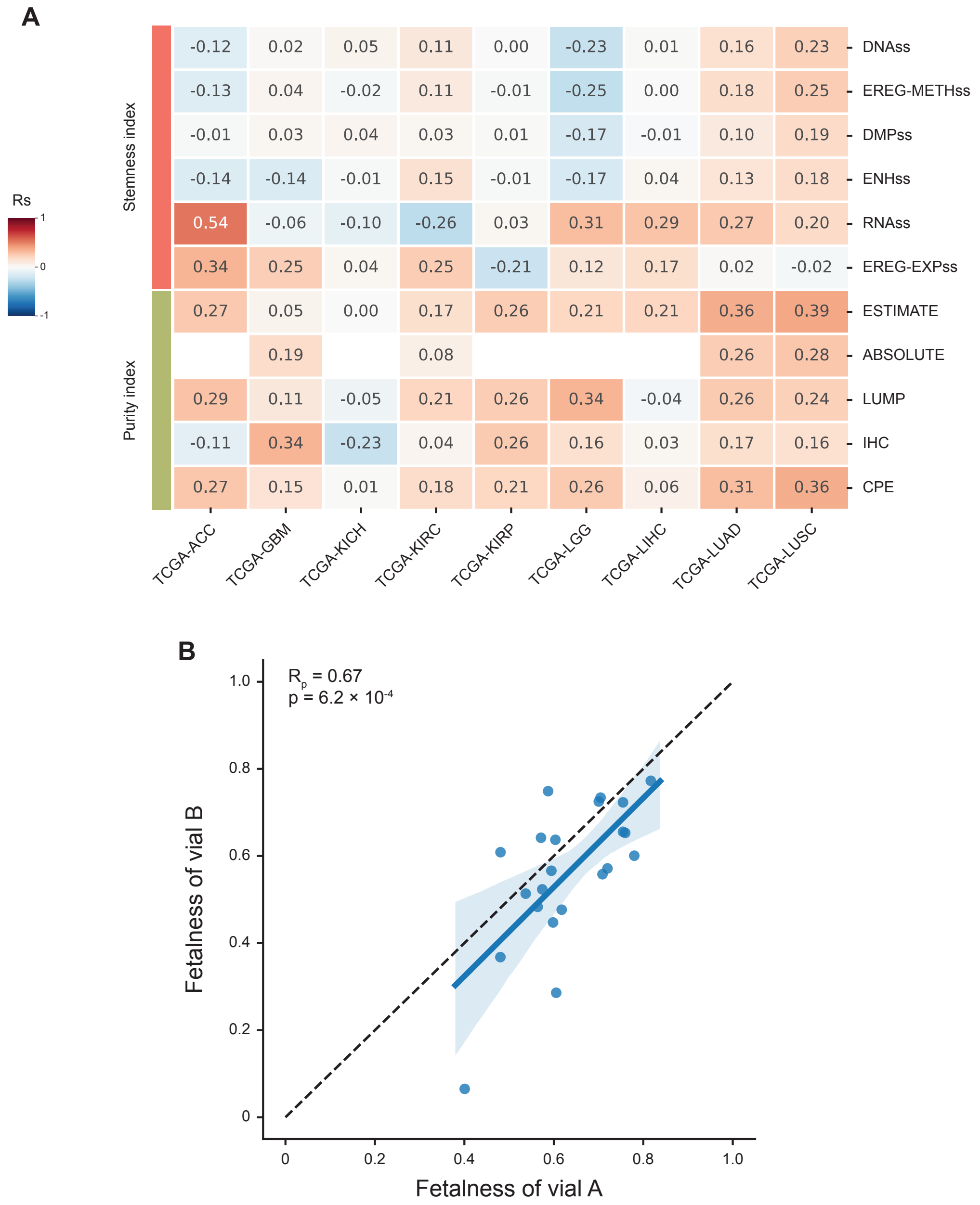
A
Rs
1
0
-1
Stemness index
Purity index
-0.12 0.02 0.05 0.11 0.00 -0.23 0.01 0.16 0.23 DNAss
-0.13 0.04 -0.02 0.11 -0.01 -0.25 0.00 0.18 0.25 EREG-METHss
-0.01 0.03 0.04 0.03 0.01 -0.17 -0.01 0.10 0.19 DMPss
-0.14 -0.14 -0.01 0.15 -0.01 -0.17 0.04 0.13 0.18 ENHss
0.54 -0.06 -0.10 -0.26 0.03 0.31 0.29 0.27 0.20 RNAss
0.34 0.25 0.04 0.25 -0.21 0.12 0.17 0.02 -0.02 EREG-EXPss
0.27 0.05 0.00 0.17 0.26 0.21 0.21 0.36 0.39 ESTIMATE
0.19 0.08 0.26 0.28 ABSOLUTE
0.29 0.11 -0.05 0.21 0.26 0.34 -0.04 0.26 0.24 LUMP
-0.11 0.34 -0.23 0.04 0.26 0.16 0.03 0.17 0.16 IHC
0.27 0.15 0.01 0.18 0.21 0.26 0.06 0.31 0.36 CPE
TCGA-ACC
TCGA-GBM
TCGA-KICH
TCGA-KIRC
TCGA-KIRP
TCGA-LGG
TCGA-LIHC
TCGA-LUAD
TCGA-LUSC
B
R_p = 0.67
p = 6.2 × 10^{-4}
Fetalness of vial B
Fetalness of vial A
0
0.2
0.4
0.6
0.8
1.0

Fig. S6. Association of the fetalness index with tumor purity and published stemness indices. (A) Heatmap showing Spearman's rank correlation coefficients between the fetal fraction and each of the 6 published stemness indices as well as the 5 tumor purity indices across primary tumor samples of 9 TCGA cancer types.

(B) Scatter plot showing the relationship between the inferred fetal fractions of 22 TCGA samples and those collected in a different vial. The colored regions around the regression lines indicate a 95% confidence interval.

Figure S7

A

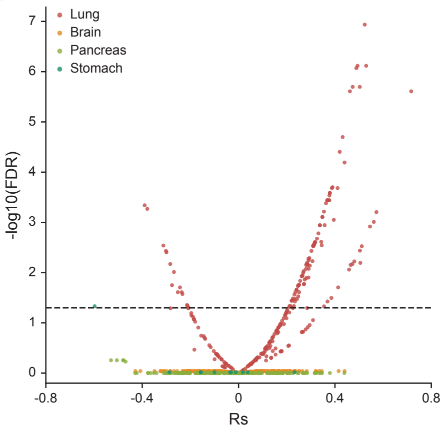
Lung
Brain
Pancreas
Stomach
-log10(FDR)
Rs
7
6
5
4
3
2
1
0
-0.8
-0.4
0
0.4
0.8

Fig. S7. Correlations of fetalness with drug response across GDSC cell lines

(A) Volcano plot of GDSC drugs with differential sensitivities associated with fetalness in cell lines of different tissue origin. The horizontal dashed line indicates a significance threshold of FDR = 0.05.
